# IRF1-mediated downregulation of PGC1α contributes to cardiorenal syndrome type 4

**DOI:** 10.1038/s41467-020-18519-0

**Published:** 2020-09-16

**Authors:** Yinghui Huang, Shaobo Wang, Jie Zhou, Yong Liu, Changhong Du, Ke Yang, Xianjin Bi, Mingying Liu, Wenhao Han, Kailong Wang, Jiachuan Xiong, Song Wang, Yue Wang, Ling Nie, Chi Liu, Daohai Zhang, Jun Gu, Chunyu Zeng, Jinghong Zhao

**Affiliations:** 1 Department of Nephrology, The Key Laboratory for the Prevention and Treatment of Chronic Kidney Disease of Chongqing, Kidney Center of PLA, Xinqiao Hospital, Army Medical University (Third Military Medical University), 400037 Chongqing, China; 2 Institute of Combined Injury, State Key Laboratory of Trauma, Burns and Combined Injury, Chongqing Engineering Research Center for Nanomedicine, College of Preventive Medicine, Army Medical University, 400038 Chongqing, China; 3 Department of Oncology and Southwest Cancer Center, Southwest Hospital, Army Medical University, 400038 Chongqing, China; 4 State Key Laboratory of Protein and Plant Gene Research, College of Life Science, Peking University, 100871 Beijing, China; 5 Department of Cardiology, Daping Hospital, Army Medical University, 400042 Chongqing, China

**Keywords:** Cardiology, Chronic kidney disease

## Abstract

Cardiorenal syndrome type 4 (CRS4) is a common complication of chronic kidney disease (CKD), but the pathogenic mechanisms remain elusive. Here we report that morphological and functional changes in myocardial mitochondria are observed in CKD mice, especially decreases in oxidative phosphorylation and fatty acid metabolism. High phosphate (HP), a hallmark of CKD, contributes to myocardial energy metabolism dysfunction by downregulating peroxisome proliferator-activated receptor gamma coactivator 1 alpha (PGC1α). Furthermore, the transcriptional factor interferon regulatory factor 1 (IRF1) is revealed as the key molecule upregulated by HP through histone H3K9 acetylation, and responsible for the HP-mediated transcriptional inhibition of PGC1α by directly binding to its promoter region. Conversely, restoration of PGC1α expression or genetic knockdown of IRF1 significantly attenuates HP-induced alterations in vitro and in vivo. These findings demonstrate that IRF1-PGC1α axis-mediated myocardial energy metabolism remodeling plays a crucial role in the pathogenesis of CRS4.

## Abstract (web summary)

The pathogenic mechanisms of cardiorenal syndrome type 4 (CRS4) remain unclear. Here, the authors identify IRF1-PGC1α axis-mediated myocardial energy metabolism remodeling as a contributor to CRS4 pathogenesis, thus providing potential new targets for reducing cardiovascular events in CKD patients.

## Introduction

Cardiovascular disease (CVD) is the most common complication of chronic kidney disease (CKD) and the leading cause of death (more than 50%) in patients with CKD^1,2^. CKD may lead to pathologic cardiac changes including left ventricular hypertrophy (LVH), diastolic dysfunction, and/or increased risk of cardiovascular events, and this condition is called cardiorenal syndrome type 4 (CRS4)^3^. Astonishingly, up to 30% of CKD patients suffer from chronic heart failure (HF)^1,2^, and about 40–50% of patients with HF have coexisting chronic renal dysfunction^4^. As reported, the risk of HF is much higher in patients with an estimated glomerular filtration rate (eGFR) <60 ml/min per 1.73 m^2^, especially in dialysis patients^2,5^. However, the mechanism of increased susceptibility to HF in CKD patients remains elusive^2,4^.

As abundant ATP is required for normal systole and diastole, a severe decrease of ATP will directly disrupt cardiac function and eventually lead to HF^6–8^. Normally, over 95% of cardiac ATP is derived from oxidative phosphorylation (OXPHO), and the remaining 5% principally comes from glycolysis. The substrate utilization in the adult heart is mainly from the oxidation of fatty acid (FAO, 70–90%), glucose and lactate (10–30%)^7^. Of note, FAO and OXPHO frequently decrease in a hypertrophic or failing heart, while glycolysis often compensatorily increases, known as myocardial energy metabolism remodeling^7^. This remodeling is considered responsible for the progression of LVH and HF^8^. A clinical study showed that a significant amelioration of cardiac function and reduction of the left ventricular mass index (LVMI) were observed in hemodialysis patients after treatment with Levocarnitine, a cofactor of FAO key enzyme carnitine palmitoyl transferase 1 (Cpt1)^9^, indicating that severe disorder of myocardial energy metabolism may exist in patients with CKD. However, there is a lack of an in-depth study on the pathogenesis of CKD-associated myocardial energy metabolism dysfunction.

Traditional risk factors of CVD are insufficient to explain the high prevalence of HF in CKD patients^10,11^, such as hypertension, hemodynamic alterations, and activation of the renin–angiotensin–aldosterone system (RAAS) and sympathetic nervous system. Therefore, increasing attention was paid to CKD-inherent risk factors, i.e. uremic toxins that accumulated in the body during disease progression. Retained uremic toxins can be divided into three groups according to physicochemical characteristics: small water-soluble compounds (molecular weight (MW) < 500 Da), middle-molecules (500 Da < MW < 32,000 Da) and protein-bound small molecules (most of them with MW < 500 Da). Remarkably, recent studies have reported that uremic toxins are not only a consequence of CKD, but also a cause of uremic symptoms, including CVD^1,2^. Several water-soluble toxins have emerged as hallmarks of CKD^12,13^ and risk factors for CVD in CKD patients^14^, such as high phosphate (HP), fibroblast growth factor 23 (FGF23), and trimethylamine-*N*-oxide (TMAO). Middle-molecules can be used to predict the severity of coronary artery disease, such as β2-microglobulin (B2M)^15^. Protein-bound toxins are associated with CKD progression and increased cardiovascular mortality, such as Indoxyl sulfate (IS) and *p*-cresyl sulfate (PCS)^16^. Despite these findings, the distinct roles of uremic toxins in the pathogenesis of CRS4 and the underlying mechanisms are not fully understood.

In this study, we demonstrate that peroxisome proliferator-activated receptor-gamma coactivator 1 alpha (PGC1α) is downregulated in the heart of CKD mice and PGC1α downregulation mediates myocardial energy metabolism remodeling that contributes to CKD-associated HF. HP is a crucial uremic toxin involved in myocardial energy metabolism dysfunction and could induce PGC1α downregulation. Moreover, the transcriptional factor interferon regulatory factor 1 (IRF1) mediates HP-induced PGC1α suppression by directly binding to its promoter region, thus generating HP-induced mitochondrial energy metabolism dysfunction and CKD-associated HF.

## Results

### Energy metabolism changes and PGC1α repression in CKD mice

**Fig. 1:**
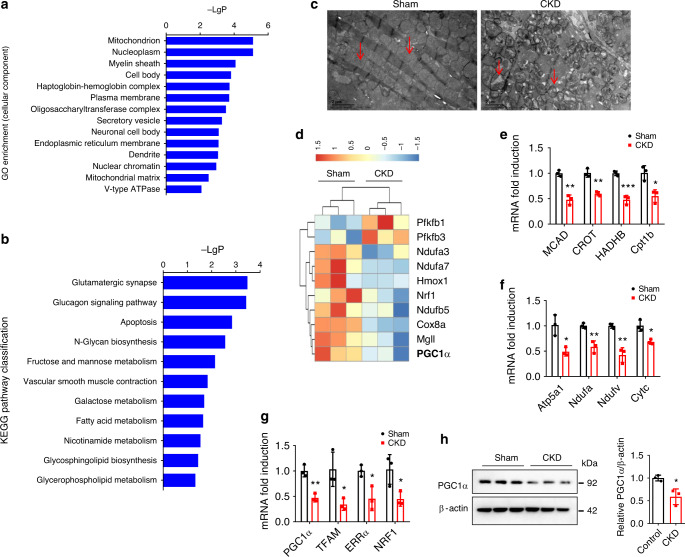
Myocardial energy metabolism dysfunction and PGC1α downregulation are observed in the heart of CKD mice. **a** GO enrichment of the significantly changed cellular components of the heart from three pairs of sham and CKD mice. **b** KEGG pathway classification of differentially expressed genes (DEG) of the heart in sham and CKD mice. **c** Representative images of transmission electron microscope (TEM) observation of mitochondria of the heart from sham and CKD mice (*n* = 3 mice per group). Scale bar, 2 μm. Red arrows point to mitochondria. **d** Heatmap of DEG (mainly PGC1α and its target genes) of mice in **a**. The color intensity represents row *Z*-score. Red color indicates highly expressed, while blue indicates lowly expressed. **e**–**g** Relative mRNA expression of FAO genes (**e**), OXPHO genes (**f**), and PGC1α and its target genes (**g**) of the heart from sham and CKD mice. **h** Western blot analysis of PGC1α expression of the heart from sham and CKD mice. Microarray data were obtained from three mice per group. Data in **a**, **b** are shown as −Log*P* and were analyzed by Fisher’s exact test, adjusted by using Benjamini–Hochberg. Data in **e**–**h** are shown as mean ± SD and were analyzed by a two-tailed unpaired *t*-test (*n* = 3). *P < 0.05, **P < 0.01, ***P < 0.001.

To broadly explore the pathogenesis of CKD-associated CVD, we first examined genome-wide transcriptional changes in the heart of CKD mice by performing microarray analysis using Affymetrix Clariom S array. Gene ontology (GO) analysis revealed that the markedly downregulated genes were focused on mitochondrion (Fig. 1a). Kyoto Encyclopedia of Genes and Genomes (KEGG)-based pathway classification of the differentially expressed genes indicated significant changes in fatty acid metabolism (Fig. 1b). Meanwhile, transmission electron microscope (TEM) observation found mitochondrial derangements, swelling, and vacuolation with disrupted cristae in cardiomyocytes of CKD mice (Fig. 1c). Notably, microarray analysis showed that PGC1α, the key regulator of mitochondrial biogenesis and energy metabolism^17,18^, and its target genes were significantly inhibited in CKD mice (Fig. 1d). Then, quantitative real-time polymerase chain reaction (qPCR) and western blot verified that PGC1α and its target genes [transcription factor A, mitochondrial (TFAM), estrogen-related receptor alpha (ERRα) and nuclear respiratory factor 1 (NRF1)], the genes related to OXPHO [ATP synthase, H+ transporting, mitochondrial F1 complex, alpha subunit 1 (Atp5a1), NADH-ubiquinone oxidoreductase alpha subunit (Ndufa), NADH-ubiquinone oxidoreductase flavoprotein (Ndufv) and cytochrome *c* (Cytc)] and the genes related to FAO [medium chain acyl-CoA dehydrogenase (MCAD), carnitine *O*-octanoyltransferase (CROT), hydroxyacyl-coenzyme A dehydrogenase (HADHB) and Cpt1b] were significantly repressed in myocardial tissues from CKD mice (Fig. 1e–h). These data indicate that CKD is associated with myocardial energy metabolism dysfunction accompanied by PGC1α downregulation in cardiomyocytes.

### Effects of HP on PGC1α expression and energy metabolism

**Fig. 2:**
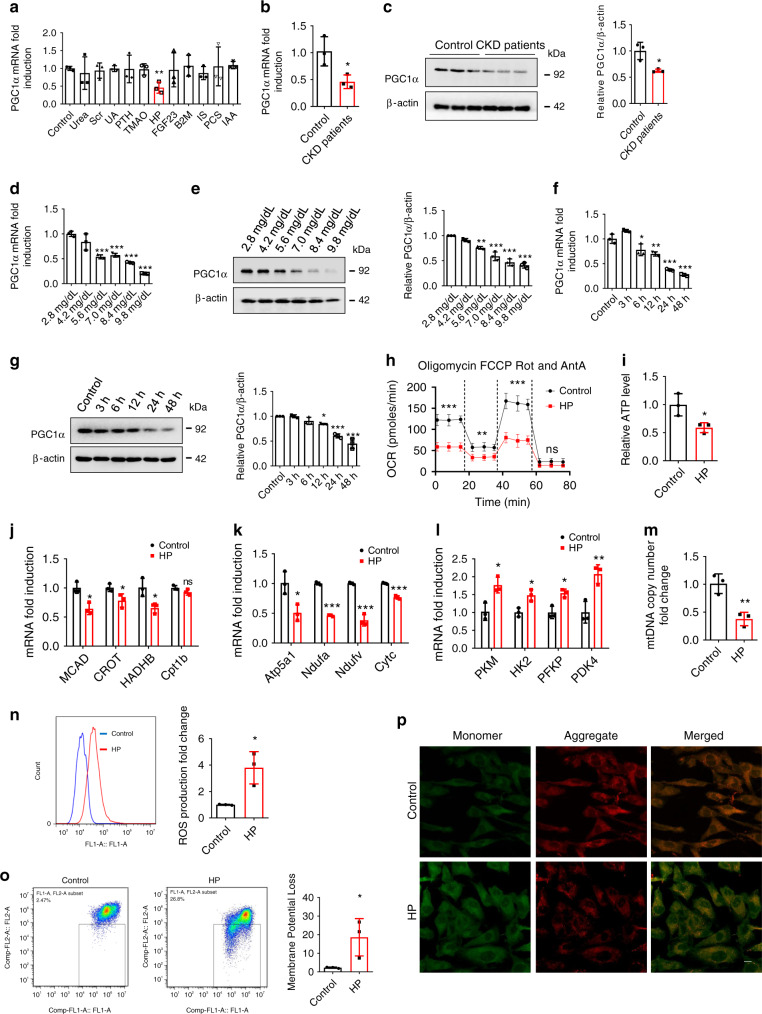
HP induces PGC1α reduction and mitochondrial energy metabolism dysfunction in vitro. **a** H9c2 cells were treated with urea (1.2 mg/ml), serum creatinine (Scr, 80 μg/ml), uric acid (UA, 80 μg/ml), parathyroid hormone (PTH, 90 ng/ml), trimethylamine-*N*-oxide (TMAO, 0.751 μg/ml), HP (84 μg/ml), FGF23 (20 ng/ml), β2-microglobulin (B2M, 20 μg/ml), indoxyl sulfate (IS, 125 μg/ml), *p*-cresyl sulfate (PCS, 47 μg/ml), and indole-3-acetic acid (IAA, 3.5 μg/ml), separately. mRNA was extracted for qPCR analysis of PGC1α expression. **b**, **c** qPCR and western blot analysis of PGC1α expression in H9c2 cells incubated with the serum of healthy donors or CKD patients for 24 h. **d**–**g** qPCR and representative western blot analysis of PGC1α expression in H9c2 cells treated with control, various doses of HP for 24 h or HP (8.4 mg/dl) for various durations. **h**–**p** Oxygen consumption rate (OCR) (**h**), relative ATP level (**i**), relative mRNA expression of FAO genes (**j**), relative mRNA expression of OXPHO genes (**k**), relative mRNA expression of glycolysis genes (**l**), relative mitochondrial DNA copy number (**m**), ROS production (**n**), and monomer and aggregate JC-1 (**o** flow cytometry; **p** representative images of laser scanning confocal microscopy. Scale bar, 10 μm) in H9c2 cells treated with control or HP for 24 h. Data are shown as mean ± SD and were analyzed by a two-tailed unpaired *t*-test (**a**–**c**, **h**–**o**) or one-way ANOVA (**d**–**g**). *n* = 3 (**a**–**g**, **i**–**p**) or *n* = 4 (**h**) biologically independent experiments. *P < 0.05, **P < 0.01, ***P < 0.001.

As the development of CKD-associated CVD is closely related to CKD-inherent risk factors^19^, we treated cardiac cells with uremic toxins to search for the causes of CKD-induced PGC1α downregulation and mitochondrial energy metabolism dysfunction. Interestingly, we observed that PGC1α expression was not significantly suppressed by urea, serum creatinine (Scr), uric acid (UA), parathyroid hormone (PTH), TMAO, FGF23, B2M, IS, PCS, or indole-3-acetic acid (IAA), whereas HP had a strong ability to inhibit PGC1α expression (Fig. 2a). Moreover, PGC1α expression was also suppressed in cardiac cells incubated with the serum of CKD patients with hyperphosphatemia (6.90 ± 1.09 mg/dl) (Fig. 2b, c). We then demonstrated that PGC1α expression was dose- and time-dependently downregulated by HP at both transcriptional and translational levels (Fig. 2d–g). To investigate the role of HP in mitochondrial energy metabolism, we treated cardiac cells with HP and found suppression of oxygen consumption rate (OCR), decrease in ATP level, downregulation of FAO and OXPHO-related genes, upregulation of glycolysis-related genes, reduction of mitochondrial DNA (mtDNA) copy number, the elevation of reactive oxygen species (ROS) production, and loss of mitochondrial membrane potential (Fig. 2h–p). In addition, similar results including downregulated PGC1α expression and energy metabolism remodeling were observed in HP-incubated primary neonatal rat ventricular myocytes (NRVMs) (Supplementary Fig. 1A–G).

To explore the relationship between HP and CKD-associated cardiac remodeling, 213 predialysis patients with eGFR of 2.00–23.30 ml/min per 1.73 m^2^ were enrolled in this study. The patients were divided into three groups according to tertiles of the serum phosphate level (2.787–10.932 mg/dl). We found that serum phosphate level was inversely associated with eGFR (*r* = −0.4387, *P* < 0.0001) (Supplementary Fig. 2A). Since ejection fraction (EF%) decreases and LVMI increases with decreasing eGFR, we used partial correlation analysis to analyze the associations of EF% and LVMI with phosphate levels in the presence of adjusted eGFR. As shown in Supplementary Fig. 2B, C, serum phosphate level was negatively associated with EF% (the partial correlation coefficient *r*_p_ = −0.3442, *P* < 0.0001), while positively correlated with LVMI (*r*_p_ = 0.3541, *P* < 0.0001). Compared with participants in the lowest phosphate tertile, those in the highest tertile had the highest LVMI (*P* = 0.0001) (Supplementary Fig. 2B) and the lowest EF% (*P* = 0.0004) (Supplementary Fig. 2C).

**Fig. 3:**
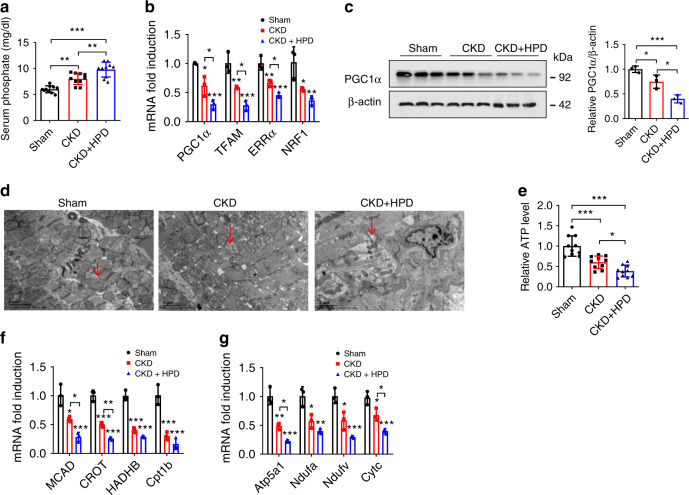
HP induces PGC1α suppression and cardiac remodeling in CKD mice. **a** Serum phosphate level in sham, CKD, and HPD-fed CKD mice for 12 weeks. *n* = 10 mice per group. **b**–**g** qPCR analysis of PGC1α and its target genes expressions (**b**), western blot analysis of PGC1α expression (**c**), representative images of TEM observation of mitochondria (**d**). Red arrows point to mitochondria, relative ATP level (**e**), relative mRNA expression of FAO genes (**f**), and relative mRNA expression of OXPHO genes (**g**) in heart tissues from the mice in **a**. *n* = 10 (**a**, **e**), or *n* = 3 (**b**–**d**, **f**, **g**). Data are shown as mean ± SD and were analyzed by one-way ANOVA. *P < 0.05, **P < 0.01, ***P < 0.001.

To evaluate the effect of HP on cardiac remodeling, H9c2 cells and NRVMs were treated with HP. We found that HP could induce cardiac hypertrophy, along with increased expressions of hypertrophic genes including atrial natriuretic factor (ANF), brain natriuretic peptide (BNP), and β-myosin heavy chain (β-MHC) (Supplementary Fig. 3A, B). Meanwhile, we fed CKD mice with high phosphate diet (HPD) for 12 weeks and found that cardiac hypertrophy and failure were aggravated in HPD-fed CKD mice (Fig. 3a and Supplementary Fig. 3C–I). Next, we confirmed that the decrease in the expressions of PGC1α and its target genes was much more prominent in HPD-fed CKD mice than in CKD mice (Fig. 3b, c). Concordantly, more apparent changes in mitochondrial derangements, ATP level, and the expressions of FAO and OXPHO-related genes were observed in HPD-fed CKD mice (Fig. 3d–g). These data suggest that HP contributes to metabolic disorders and cardiac remodeling in vivo.

### PGC1α protects against HP-induced pathological changes

**Fig. 4:**
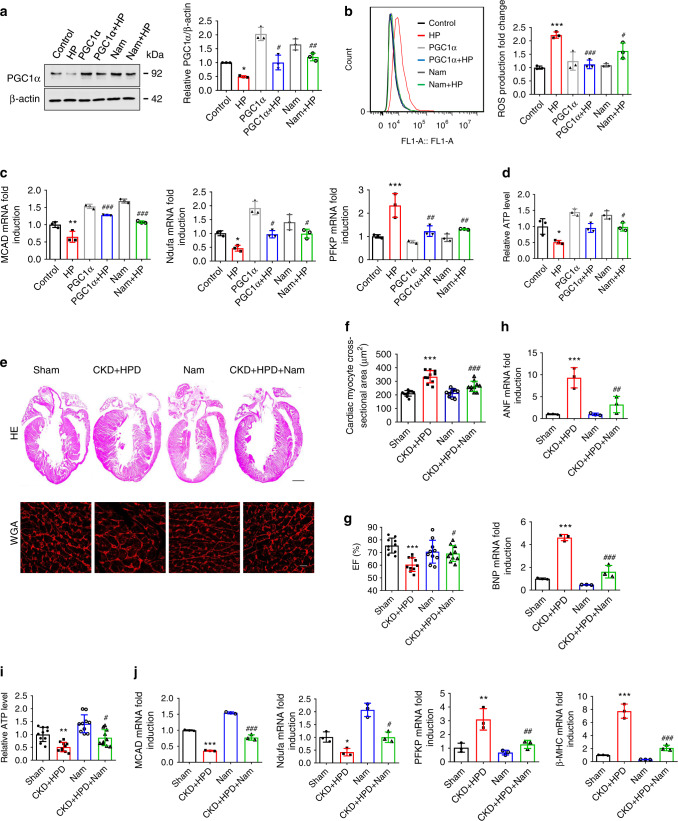
Restoration of PGC1α expression protects against HP-induced mitochondrial energy metabolism dysfunction and cardiac remodeling in both cardiac cells and CKD mice. **a**–**d** H9c2 cells were transfected with PGC1α overexpression plasmids or pretreated with Nam, and then treated with control or HP for representative western blot analysis of PGC1α expression (**a**) ROS production (**b**) relative mRNA expression of MCAD (FAO), Ndufa (OXPHO), and PFKP (glycolysis) (**c**), and relative ATP level (**d**). **e** Representative gross pathology (HE staining, upper panel. Scale bar, 1 mm) and WGA staining (lower panel. Scale bar, 10 μm) of heart sections from sham, HPD-fed CKD, Nam, and HPD-fed CKD mice intraperitoneally injected with Nam for 12 weeks. *n* = 10 mice per group. **f** Cross-sectional surface area of individual cardiac myocytes from the mice in the lower panel of (**e**). **g**–**j** Echocardiography of EF% (**g**), qPCR analysis of the mRNA expression of hypertrophic genes (**h**), relative ATP level (**i**), relative mRNA expression of metabolic genes (**j**) of heart lysates from the mice in (**e**). *n* = 3 (**a**–**d**, **h**, **j**), or *n* = 10 (**e**–**g**, **i**). Data are shown as mean ± SD and were analyzed by one-way ANOVA. *P < 0.05, **P < 0.01, ***P < 0.001 versus control or sham. ^#^*P* < 0.05, ^##^*P* < 0.01, ^###^*P* < 0.001 versus HP or CKD + HPD.

As the preservation of PGC1α expression has therapeutic potential for HF^18,20^, we sought to determine whether restoration of PGC1α expression could rescue HP-induced changes. As expected, both PGC1α overexpression and nicotinamide (Nam, a key effector of PGC1α^17,21^) treatment rescued HP-induced disturbance of PGC1α expression, ROS production, metabolic genes expression, ATP level, and cardiomyocyte hypertrophy (Fig. 4a–d and Supplementary Fig. 4A). Concordantly, cardiac hypertrophy, heart failure, and myocardial energy metabolism remodeling in HPD-fed CKD mice were significantly ameliorated by Nam treatment (Fig. 4e–j and Supplementary Fig. 4B–D).

### HP regulates PGC1α expression and energy metabolism via IRF1

**Fig. 5:**
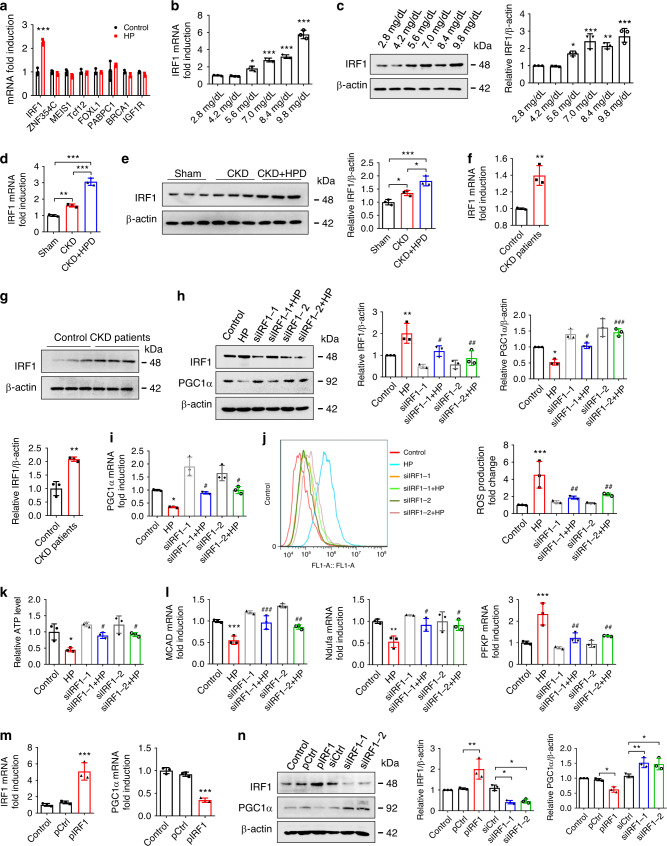
HP disturbs PGC1α expression and energy metabolism in an IRF1-dependent manner. **a** qPCR analysis of the expression of the predicted transcriptional factors in H9c2 cells treated with control or HP for 24 h. **b**, **c** qPCR and representative western blot analysis of IRF1 expression in H9c2 cells treated with control or various doses of HP for 24 h. **d**–**g** qPCR and representative western blot analysis of IRF1 expression in heart lysates from sham, CKD, and HPD-fed CKD mice for 12 weeks (**d**, **e**) or in H9c2 cells incubated with the serum of healthy donors or CKD patients for 24 h (**f**, **g**). **h**–**l** H9c2 cells were transfected with two pairs of siRNA against IRF1, and then treated with control or HP for 24 h. Cells were collected for detection of IRF1 and PGC1α protein expressions (**h**), PGC1α mRNA expression (**i**), ROS production (**j**), relative ATP level (**k**), and mRNA expression of the metabolic genes (**l**). **m** qPCR analysis of IRF1 and PGC1α expressions in H9c2 cells transfected with control plasmids (pCtrl) or IRF1 overexpression plasmids (pIRF1). **n** Representative western blot analysis of IRF1 and PGC1α expressions in H9c2 cells transfected with pCtrl, pIRF1, control siRNA (siCtrl), and two pairs of siRNA against IRF1 (siIRF1), separately. *n* = 3 biologically independent experiments (**a**–**n**). Data are shown as mean ± SD and were analyzed by a two-tailed unpaired *t-*test (**a**, **f**, **g**) or one-way ANOVA (**b**–**e**, **h**–**n**). *P < 0.05, **P < 0.01, ***P < 0.001 versus control. ^#^*P* < 0.05, ^##^*P* < 0.01, ^###^*P* < 0.001 versus HP.

Then, we tried to explore the mechanism underlying HP-induced PGC1α downregulation. Bioinformatics analysis predicted 36 transcriptional factors that could bind to PGC1α promoter region, 8 of which [IRF1, zinc finger protein 354C (ZNF354C), Meishomeobox 1 (MEIS1), transcription factor 12 (Tcf12), forkhead box L1 (FOXL1), poly(A) binding protein cytoplasmic 1 (PABPC1), breast cancer type 1 susceptibility protein (BRCA1), and insulin-like growth factor 1 receptor (IGF1R)] were associated with cardiac hypertrophy. Interestingly, IRF1 was the only upregulated transcriptional factor in HP-treated cells (Fig. 5a–c). Moreover, the expression level of IRF1 was much higher in H9c2 cells than that of other IRF family members unable to be regulated by HP (Supplementary Fig. 5A, B). Of note, a more remarkable increase of IRF1 expression was observed in HPD-fed CKD mice than in control or CKD mice (Fig. 5d, e). Furthermore, IRF1 expression was also upregulated in H9c2 cells incubated with CKD patients’ serum (Fig. 5f, g) and in NRVMs after HP treatment (Supplementary Fig. 5C, D). Considering the crucial role of IRF1 in cardiac hypertrophy^22^, we hypothesized that HP might suppress PGC1α expression via upregulating IRF1 expression. To test this hypothesis, two pairs of different siRNAs against IRF1 (siIRF1) were transfected into H9c2 cells, and we found that both of them could rescue the HP-induced disturbance of PGC1α expression, ROS production, ATP level, metabolic gene expression and cardiomyocyte hypertrophy (Fig. 5h–l and Supplementary Fig. 5E, F). Moreover, PGC1α was downregulated in IRF1 overexpression plasmids-transfected cells (Fig. 5m, n), and increased expressions of PGC1α, PGC1α target genes, and FAO- and OXPHO-related genes were detected in IRF1^−/−^ mice (Supplementary Fig. 5G–K). All these data support the hypothesis that IRF1 may mediate the effect of HP on PGC1α downregulation.

### IRF1 directly binds to PGC1α promoter region for inhibition

**Fig. 6:**
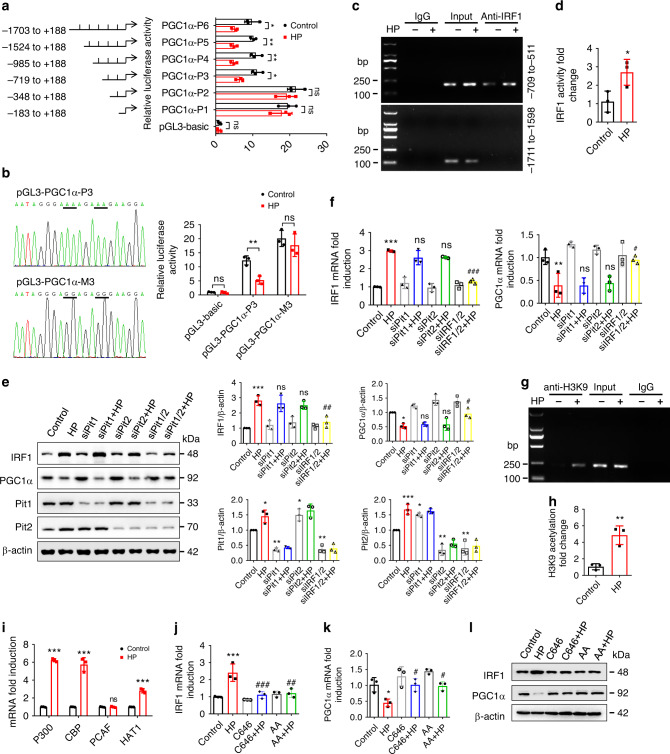
IRF1 inhibits PGC1α expression via directly binding to its promoter region. **a** After being co-transfected with pRL-TK vector and pGL3-basic or recombinant reporter plasmids containing various fragments of PGC1α promoter region, H9c2 cells were treated with control or HP and then harvested for dual-luciferase reporter assay. The firefly luciferase activity was normalized against Renilla activity. **b** H9c2 cells were co-transfected with pRL-TK vector and pGL3-PGC1α-P3 or pGL3-PGC1α-M3 (containing the mutant bases of pGL3-PGC1α-P3 underlined in the sequencing results), treated with control or HP and harvested for luciferase assay. **c**, **d** H9c2 cells were treated with control or HP for 24 h and harvested for ChIP assay. IRF1 antibody was immunoprecipitated with chromatin DNA fragments, taking IgG as a negative control. The precipitated DNA was amplified by PCR **c** and qPCR **d** using primers that cover the PGC1α promoter region (−709 to −511), taking primers that cover the region (−1711 to −1598) as a negative control. **e**, **f** qPCR and representative western blot analysis of IRF1 and PGC1α expressions in cells treated with siPit1, siPit2, or both in the presence or absence of HP. **g**, **h** H9c2 cells were treated with control or HP, and H3K9 acetylation was assayed using ChIP. **i** qPCR analysis of histone acetyltransferase genes expression in H9c2 cells treated with control or HP. **j**–**l** qPCR and western blot analysis of IRF1 and PGC1α expressions in cells treated with a P300/CBP inhibitor (C646, 10 μM) or a pan-histone acetyltransferase inhibitor (anacardic acid, AA,10 μM) in the presence or absence of HP. Data are shown as mean ± SD and were analyzed by a two-tailed unpaired *t*-test (**a**–**d**, **h**, **i**) or one-way ANOVA (**e**, **f**, **j**, **k**). *n* = 3 biologically independent experiments (**a**, **b**, **d**–**f**, **h**–**k**). *P < 0.05, **P < 0.01, ***P < 0.001 versus control. ^#^*P* < 0.05, ^##^*P* < 0.01, ^###^*P* < 0.001, ns: no significance versus HP.

Next, we explored the mechanism by which IRF1 regulates PGC1α expression. We first found that the mRNA stability and protein degradation of PGC1α were not significantly affected by HP (Supplementary Fig. 6). Of note, bioinformatics analysis showed that IRF1 had the potential to bind to the PGC1α promoter region (Supplementary Table 1), hinting that IRF1 may inhibit PGC1α expression via directly binding to its promoter region. To prove this speculation, six truncated PGC1α reporter plasmids were transfected into H9c2 cells. As shown in Fig. 6a, HP markedly inhibited the luciferase activities of the reporter plasmids pGL3-PGC1α-1703, pGL3-PGC1α-1524, pGL3-PGC1α-985 and pGL3-PGC1α-719 (rather than pGL3-PGC1α-348 or pGL3-PGC1α-183), indicating an IRF1 response element within the sequence containing −719 to −348 nucleotides relative to the transcriptional start site. Therefore, the putative binding sequence should be the portion containing −632 to −612 nucleotides (TCCTTCTTTCTTTTCCCTATT) according to bioinformatics analysis (Supplementary Table 1). We then demonstrated that mutation of the IRF1 response element in this putative binding sequence (−632 to −612) almost abolished HP-regulated PGC1α promoter activity (Fig. 6b), while a mutation in the sequence containing −974 to −954 nucleotides was unable to rescue HP-mediated changes (Supplementary Fig. 7). ClustalW analysis showed that the sequence containing −632 to −612 nucleotides in the PGC1α promoter region was highly conserved among 8 different mammals (Supplementary Table 2). To determine whether IRF1 can bind to the PGC1α promoter region directly, chromatin immunoprecipitation (ChIP) assay was performed. The assay revealed direct binding of IRF1 to the PGC1α promoter region (−709 to −511), which could be enhanced by HP (Fig. 6c, d), compared with a negative control.

Subsequently, we attempted to gain mechanistic insight into the regulation of IRF1 expression by HP. To investigate how phosphate is transported into cardiomyocytes, we screened the expressions of phosphate transporters and found that solute carrier family 20 member 1 (SLC20A1, Pit1) and SLC20A2 (Pit2) were highly expressed in both H9c2 cells and NRVMs (Supplementary Fig. 8A). We then revealed that Pit1 and Pit2 could be upregulated by HP (Fig. 6e), while simultaneous knockdown of Pit1 and Pit2 could evidently decrease intracellular phosphate level and abrogate HP-induced IRF1 upregulation, PGC1α downregulation and cardiomyocyte hypertrophy (Fig. 6e, f and Supplementary Fig. 8B–E), indicating that the action of HP depends on the transporters Pit1 and Pit2.

To further uncover the mechanisms underlying the regulation of IRF1 expression by HP, we examined the contributions of DNA methylation and histone acetylation, as HP has the potential to regulate DNA methylation and histone acetylation^23^. Methylation-specific PCR (MSP) using two different primers showed no significant change of DNA methylation in the IRF1 promoter region after HP treatment (Supplementary Fig. 9A, B). However, more acetylated H3K9 (Fig. 6g, h), rather than H4K12 (Supplementary Fig. 10), was found to bind to the IRF1 promoter region after HP treatment, suggesting that histone H3K9 acetylation occurred. We then demonstrated that HP-induced expressions of histone acetyltransferases [E1A binding protein p300 (P300), CREB binding protein (CBP), and histone acetyltransferase 1 (HAT1)] (Fig. 6i). The inhibition of H3K9 acetylation by P300/CBP inhibitor (C646) or pan-HAT inhibitor (anacardic acid, AA) significantly attenuated HP-induced IRF1 upregulation and PGC1α downregulation (Fig. 6j–l). These data further validate the role of H3K9 acetylation in HP-regulated IRF1 transcription.

### *kl/kl* mice display similar changes to HP-induced effects

**Fig. 7:**
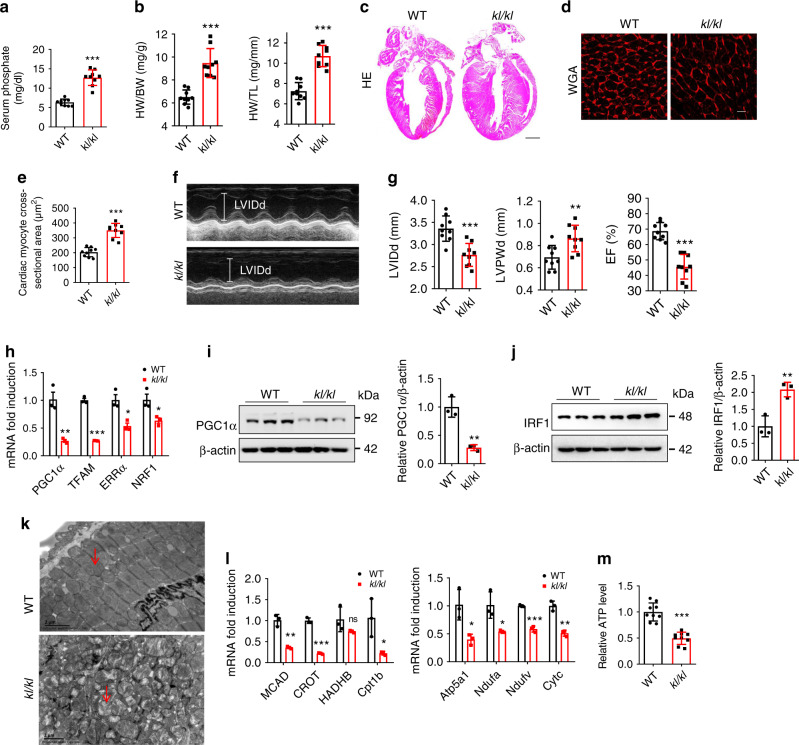
Klotho deficiency leads to hyperphosphatemia, LVH, and heart failure. **a** Serum phosphate levels of wild type (WT) and *kl/kl* mice. *n* = 9 mice per group. **b** The ratios of heart weight (HW) to body weight (BW), and HW to tibial length (TL) of mice in **a**. **c**, **d** Representative gross pathology (**c** HE staining, scale bar, 1 mm) and WGA staining (**d** scale bar, 10 μm) of heart sections from WT and *kl/kl* mice. **e** Cross-sectional surface area of individual cardiac myocytes from mice in **d**. **f**, **g** Echocardiography of LVIDd, LVPWd, and EF% in WT and *kl/kl* mice. **h**–**k** qPCR analysis of PGC1α and its target genes (**h**), western blot analysis of PGC1α (**i**), and IRF1 (**j**) expressions, representative images of TEM observation of mitochondria (**k**). Red arrows point to mitochondria. Scale bar, 2 μm, relative mRNA expression of metabolic genes (**l**), and relative ATP level **m** in the heart samples from WT and *kl/kl* mice. *n* = 9 (**a**, **b**, **e**, **g**, **m**), or *n* = 3 (**h**–**l**). Data are shown as mean ± SD and were analyzed by a two-tailed unpaired *t*-test (**a**, **b**, **e**, **g**–**j**, **l**–**m**). *P < 0.05, **P < 0.01, ***P < 0.001.

Given the serological characteristics of hyperphosphatemia that accompanied CKD and CVD in *Klotho* mutant (*kl/kl*) mice^24,25^, we then wondered whether the HP-induced changes in energy metabolism remodeling could also be detected in cardiomyocytes in *kl/kl* mice. As anticipated, the serum phosphate level was significantly higher in *kl/kl* mice (12.72 ± 1.99 mg/dl) than in wild type (WT) mice (6.35 ± 0.86 mg/dl) (Fig. 7a), even higher than in HPD-fed CKD mice (9.79 ± 1.50 mg/dl) (Fig. 3a). Notably, cardiac hypertrophy and failure were also observed in *kl/kl* mice (Fig. 7b–g), accompanied by decreased PGC1α expression, increased IRF1 expression, mitochondrial dysfunction, and energy metabolism remodeling (Fig. 7h–m). In addition, a higher serum phosphate level along with dysregulated IRF1–PGC1α axis and energy metabolism remodeling were observed in adenine-induced CKD mice (Supplementary Fig. 11A–K), further confirming the role of hyperphosphatemia in CKD-associated HF and involved mechanism.

Studies have shown that mutual interactions exist among klotho, FGF23, PTH, and Vitamin D (VitD) during their regulation of phosphate levels in vivo^24,25^. Since hyperphosphatemia and high levels of FGF23 were both detected in *kl/kl*, CKD, and HPD-fed mice (Supplementary Fig. 12A–C), we then explored the effect of FGF23 on HP-mediated changes. Interestingly, an additive effect between FGF23 and HP was observed in inducing cardiac hypertrophy and energy metabolism remodeling (Supplementary Fig. 13A–D). However, inhibitors of neither FGF23 nor FGF23 receptor (FGFR) had effects on the expressions of IRF1 and PGC1α (Supplementary Fig. 13E–O), reflecting that HP-mediated alterations in the IRF1–PGC1α axis and the subsequent energy metabolism remodeling are independent of FGF23.

### Genetic knockdown of IRF1 protects against HPD-induced HF

**Fig. 8:**
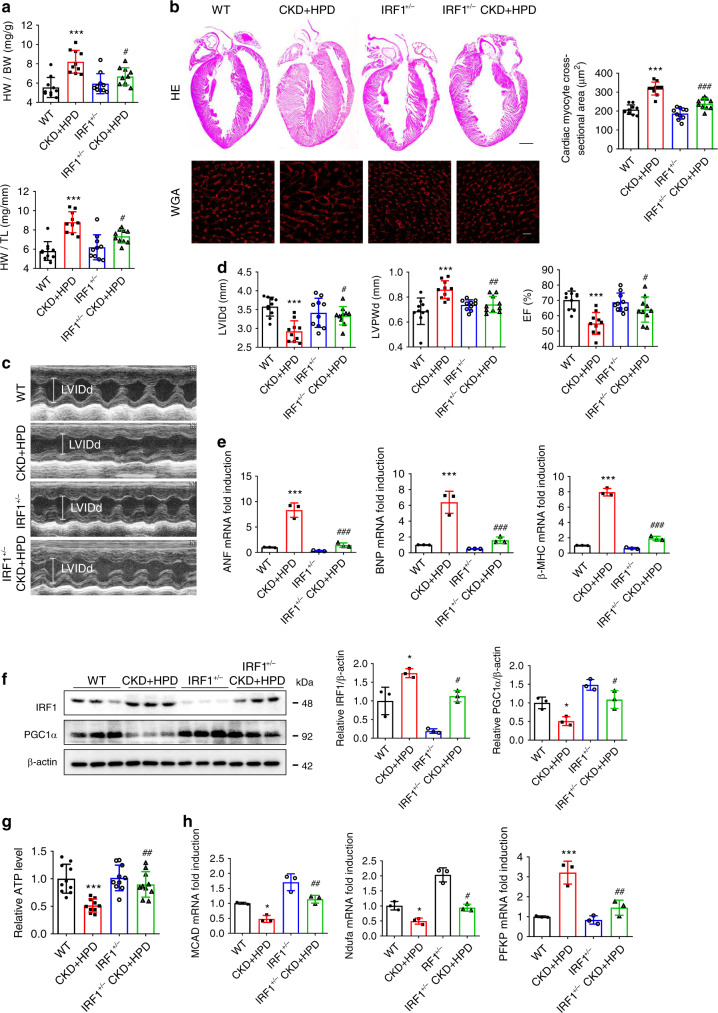
Genetic knockdown of IRF1 protects against HPD-induced HF in CKD mice. **a** The ratios of HW to BW and HW to TL of WT, HPD-fed WT CKD, IRF1^+/−^, and HPD-fed IRF1^+/−^ CKD mice for 12 weeks. *n* = 10 mice per group. **b** Representative gross pathology of heart sections (HE staining, upper panel, scale bar, 1 mm), WGA staining of the left ventricle of heart sections (lower panel, scale bar, 10 μm), and cross-sectional surface area of individual cardiac myocytes from mice in **a**. **c**, **d** Echocardiographic detection of LVIDd, LVPWd, and EF% in mice in **a**. **e**–**h** qPCR analysis of hypertrophic genes expression (**e**), western blot analysis of IRF1 and PGC1α expressions (**f**), relative ATP level (**g**), and relative mRNA expression of metabolic genes (**h**) of heart lysates from the mice in **a**. β-actin was taken as the loading control. *n* = 10 (**a**–**d**, **g**), or *n* = 3 (**e**, **f**, **h**). Data are shown as mean ± SD and were analyzed by one-way ANOVA. *P < 0.05, **P < 0.01, ***P < 0.001 versus WT. ^#^*P* < 0.05, ^##^*P* < 0.01, ^###^*P* < 0.001 versus CKD + HPD.

Finally, we verified the role of IRF1 in HP-induced changes in vivo. As shown in Fig. 8, cardiac hypertrophy and failure, PGC1α downregulation, and energy metabolism remodeling in HPD-fed CKD mice were significantly ameliorated by knocking down IRF1 expression in IRF^+/−^ mice (Fig. 8a–h). To better clarify the direct role of IRF1 in HP-induced alterations, we fed IRF1 knockout (IRF1^−/−^) mice with HPD for 12 weeks and found that HPD-induced cardiac hypertrophy, HF, downregulated PGC1α expression and energy metabolism remodeling were significantly attenuated in IRF1^−/−^ mice (Supplementary Fig. 14A–J). These results demonstrate that IRF1 not only has a distinctive role in HP-induced HF, but also is a potential target for the treatment of CKD-associated CVD.

## Discussion

**Fig. 9:**
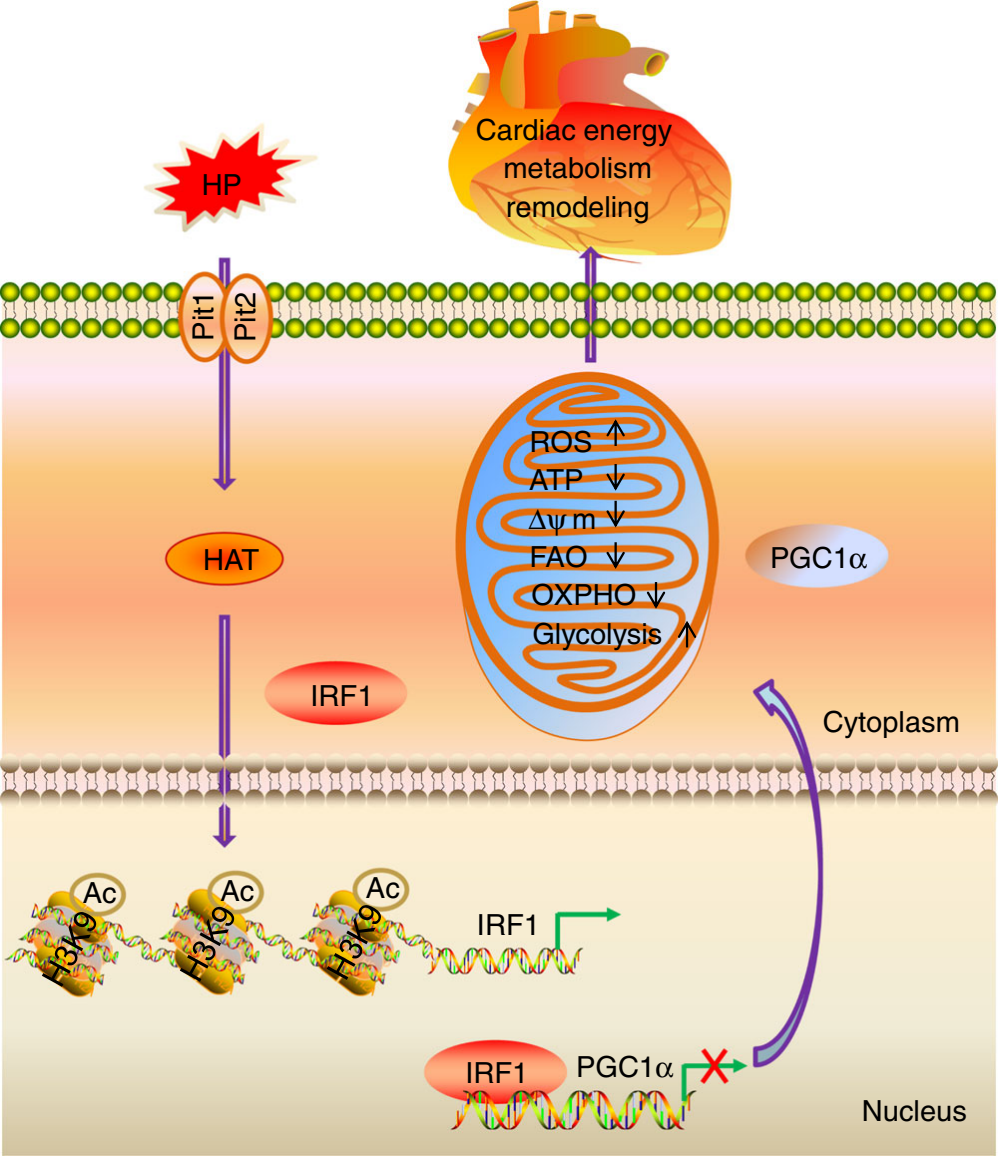
A proposed model of the mechanism underlying myocardial energy metabolism remodeling and HF induced by HP. High phosphate (HP) enters cardiomyocytes via Pit1 and Pit2, and induces IRF1 expression through acetylating histone H3K9 in the *IRF1* promoter region, during which histone acetyltransferases (HAT) are involved. HP-induced IRF1 inhibits PGC1α expression via directly binding to its promoter region (−632 to −612). PGC1α downregulation contributes to HP-induced mitochondrial dysfunction and myocardial energy metabolism remodeling. Restoration of PGC1α expression or knockdown of IRF1 protects against CKD-associated myocardial energy metabolism remodeling and HF.

CRS4, a prevalent complication of CKD with a high incidence, has emerged as a leading cause of death in CKD patients^1,2,5^, but the pathogenic mechanism remains elusive. In the present study, we demonstrate that PGC1α downregulation and consequent myocardial energy metabolism remodeling contribute to CKD-associated HF, during which hyperphosphatemia has a pivotal role. Mechanistically, HP disturbs PGC1α expression through epigenetic regulation of IRF1. Pharmacological restoration of PGC1α expression or genetic knockdown of IRF1 protects against CKD-associated myocardial energy metabolism remodeling and HF (Fig. 9).

Multiple risk factors contribute to HF, including hypertension, hemodynamic alterations, RAAS activation, and sympathetic nervous system activation, but they are insufficient to explain the high prevalence of HF in CKD patients^10,11^. In this study, microarray analysis and TEM observation identified both functional and morphological changes of mitochondria in myocardial tissues of CKD mice. As reported, HF mostly occurs in CKD patients at stages 4–5, especially in dialysis patients^2,26^. As the accumulation of uremic toxins is the most prominent and specific feature of CKD patients^2,26^, we screened CKD-specific toxins and identified HP as the toxin able to induce energy metabolism dysfunction. As known, phosphorus is an essential element for energy production and transfer^27^. However, long-term accumulation of phosphate is detrimental to health^27^. As reported in a community-based study enrolling 4494 participants without CVD, each 20% increase in dietary phosphate intake was associated with an estimated 1.1 g greater left ventricular mass (LVM)^28^. In contrast, phosphate binders can prevent aortic stiffness, diastolic dysfunction, and LVH in CKD mice^29^, and are also able to improve the survival rate in CKD patients^30^. However, the mechanisms underlying the harmful effects of hyperphosphatemia remain unclear^12^.

It is known that the functional and morphological changes of mitochondria affect ATP production in cardiomyocytes, thereby disrupting cardiac function and eventually leading to HF^6–8^. In the present study, we found that HP treatment led to mitochondrial energy metabolism remodeling via inhibiting the expression of PGC1α, a master regulator of mitochondrial biogenesis and energy metabolism^17,18,20^. Growing evidence suggests that downregulation of PGC1α may initiate the switch from fatty acid oxidation to glucose utilization^18,31^, especially in the process of chronic heart disease^31,32^. In this study, to our best knowledge, we are the first to discover that HP suppressed PGC1α expression both in vitro and in vivo. We also found that PGC1α suppression induced dysregulation of FAO-associated genes possibly through affecting NRF1, a target gene of PGC1α, and an important regulator of OXPHO and FAO^33,34^ (Supplementary Fig. 15A–D). Interestingly, although many FAO-related genes are controlled by peroxisome proliferator-activated receptor α (PPARα), of which PGC1α is a coactivator, we found no significant change in PPARα expression after HP treatment (Supplementary Fig. 15E, F), further reflecting that HP-induced dysregulation of FAO-related genes is due to PGC1α downregulation. On the other hand, the restoration of PGC1α expression significantly rescued HP-induced cardiomyocyte hypertrophy and energy metabolism dysfunction, suggesting that PGC1α is a potential therapeutic target for CKD-associated HF^18,20,31^.

Normally, HP is unable to directly regulate PGC1α expression at the transcriptional level. By bioinformatics analysis, we revealed that IRF1 was responsible for the downregulation of PGC1α in HP-treated cardiomyocytes. IRF1 is known as an important regulator of genes involved in immune responses, inflammatory reactions, and tumor development^35^. IRF1 has a repressor domain, which contributes to its growth inhibitory activity^35^. As reported, IRF1 can directly bind to the IRF response element in the promoter region of the target gene to negatively regulate its transcription^36^. Here, we found that PGC1α was a target gene of IRF1 and IRF1 suppressed PGC1α expression via directly binding to its promoter region (−632 to −612). This negative regulation of PGC1α by IRF1 was further confirmed by the finding that knockdown of IRF1 significantly rescued HP-induced downregulation of PGC1α, mitochondrial energy metabolic dysfunction, and cardiac hypertrophy. Of note, IRF1 has been reported to be involved in CVDs by activating inducible nitric oxide synthase (iNOS) and affecting the interleukin-18 (IL-18)-osteopontin (OPN) signaling pathway^22,37^. In this study, we demonstrate that the increase of IRF1 expression can induce energy metabolism remodeling by directly downregulating PGC1α in cardiomyocytes, thus providing new insight into the mechanisms of IRF1-induced cardiac hypertrophy. In addition, although some other members of the IRFs family were reported to be associated with HF^38^, their expressions were not significantly changed after HP treatment, reflecting that IRF1 is a distinctive target of HP.

As known, HP is capable of inducing epigenetic modifications, including DNA methylation and histone acetylation^23,39^, which mediate the effects of HP, such as fibrotic fibroblast activation and osteogenic transdifferentiation^39,40^. As cardiac hypertrophy and HF could be rescued by inhibition of DNA methylation or histone acetylation^41,42^, methylation and acetylation may also have important roles in the pathogenesis of cardiac hypertrophy. However, the underlying mechanisms are not fully uncovered, and it is unknown whether HP can induce epigenetic modifications of IRF1. In this study, the effect of HP on the methylation of IRF1 was excluded, because no significant change of methylation in CpG islands of IRF1 promoter was detected after HP treatment. Instead, we detected acetylation of histone H3K9 in the IRF1 promoter region, and increased expression of HAT was observed after HP treatment. Moreover, pretreatment with HAT inhibitors could significantly attenuate HP-induced IRF1 upregulation and PGC1α downregulation. Therefore, HP-induced upregulation of IRF1 and subsequent downregulation of PGC1α are probably due to the ability of HP to induce histone acetylation.

Phosphate is transported into cells primarily through three transporter families: type I (SLC17 family), type II (SLC34 family), and type III (SLC20 family) transporters^43^. In this study, after screening we found that type III phosphate transporters, Pit1 and Pit2, were highly expressed in cardiomyocytes and also upregulated by HP. Interestingly, we found that a compensatory effect existed between Pit1 and Pit2 in cardiomyocytes, consistent with a previous report^43^. Hence, it is necessary to knock down Pit1 and Pit2 synchronously to inhibit the effect of HP on the IRF1–PGC1α axis and cardiomyocyte hypertrophy, which was confirmed in our study.

Although hyperphosphatemia, a typical serological feature of CKD patients, acts as an independent risk factor for cardiovascular death^14,27^, we are aware that altering phosphate homeostasis in vivo may lead to changes in FGF23, PTH, Klotho, and VitD, which can also contribute to myocardial hypertrophy and energy metabolism changes^24,25^. Therefore, it is difficult to exclude the roles of the above minerals and hormones in HP-induced effects in vivo. Previous studies have suggested that HP may also have an indirect effect on the cardiovascular system by elevating FGF23^44^, which can promote cardiac hypertrophy through activation of the FGFR-calcineurin-nuclear factor of the activated T cells (NFAT) signaling pathway^12^. In this study, we found that HP-mediated alterations in the IRF1–PGC1α axis were independent of the FGF23-FGFR signaling pathway. However, an additive effect between FGF23 and HP on inducing cardiac hypertrophy and energy metabolism changes was observed, indicating that end-stage renal disease (ESRD) or dialysis patients with hyperphosphatemia and high FGF23 levels may suffer more from myocardial hypertrophy. Because hyperphosphatemia can also increase the secretion of FGF23, it is important to control hyperphosphatemia in CKD patients to alleviate cardiac injury.

In addition, phosphate can interact with calcium, pyrophosphate, and fetuin-A, possibly contributing to CVD through precipitation^45,46^. Although we demonstrated that pyrophosphate or fetuin-A had no influence on the HP-mediated IRF1–PGC1α axis (Supplementary Fig. 16), the harmful effect of HP on the cardiovascular system via interaction with calcium, pyrophosphate, and fetuin-A cannot be neglected. Despite the complex function of phosphate, the genetic knockdown of IRF1 was able to, at least partially, rescue HP-mediated cardiac energy metabolism remodeling and hypertrophy in vitro and in vivo, suggesting a distinctive and important role of the IRF1–PGC1α axis in CRS4.

In summary, our findings demonstrated that HP-induced alterations in the IRF1–PGC1α signaling pathway had an important role in the pathogenesis of CRS4 and restoration of PGC1α expression or knockdown of IRF1 improved energy metabolic dysfunction and HF under uremic milieu. Our study suggests that control of hyperphosphatemia or targeted intervention on HP-mediated IRF1 elevation and PGC1α downregulation could be a potential therapy for reducing the risk of cardiovascular death in CKD patients.

## Methods

### Patient samples collection

A total of 213 predialysis CKD patients (aged 18–80 years old) were enrolled in this study (Department of Nephrology of Xinqiao Hospital, Chongqing, China). The pre-established exclusion criteria were diabetes, pregnancy, HIV, polycystic kidney disease, renal cancer, and recent immunosuppressive therapy. The basic information and laboratory parameters including serum inorganic phosphate and eGFR of the enrolled patients were collected. Echocardiograms were performed on the first day of hospitalization using two-dimensional M-mode. EF%, ventricular dimension and wall thickness were detected at end-diastole and end-systole using IE33–5S (Philips Medical System). LVMI was calculated by indexing left ventricular mass to height^2^. Serum samples of three healthy donors and three CKD patients at stage 5 were collected for later experiments. Informed written consent was provided by all participants. All human studies, including collection and use of samples, were approved by the Ethics Committee of Xinqiao Hospital of the Army Medical University (No. 2018-006-01), and the study design and conduct complied with all relevant regulations regarding the use of human study participants and was conducted in accordance to the criteria set by the Declaration of Helsinki, and the study is compliant with the guidance of the Ministry of Science and Technology (MOST) for the Review and Approval of Human Genetic Resources.

### Animal treatment

IRF1 knockout mice were obtained from Jackson Laboratory (Bar Harbor, ME, USA), which were backcrossed to C57BL/6J background to produce heterozygous IRF1^+/−^ mice. *kl/kl* mice were provided by Jun Gu (State Key Laboratory of Protein and Plant Gene Research, College of Life Science, Peking University, Beijing, China), and the mice were backcrossed to C57BL/6J background for more than 6 generations. C57BL/6J and Balb/c mice were purchased from Beijing Huafukang Bioscience (Beijing, China). Male Balb/c, WT C57BL/6J and IRF1^+/−^ mice of 8-week-old were inflicted with 2/3 electrocoagulation of the right renal cortex (the lower and upper thirds of the kidney) and received left total nephrectomy two weeks later to construct 5/6 nephrectomy CKD model, which is in accordance with the clinical manifestations of human ESRD patients and described in multiple reports including our own studies^11,47^. Mice underwent laparotomy without kidney operation served as a sham group. Then the mice were randomly separated into the following groups and were blinded to the investigators who helped collect samples. (1) Sham and CKD Balb/c mice fed with normal diet for 8 weeks. (2) Sham, CKD, CKD + HPD (fed with 2% HPD) Balb/c mice for 12 weeks. (3) Sham, CKD + HPD, Nam (intraperitoneally injected with 400 mg/kg/day. Sigma, St. Louis, MO, USA), CKD + HPD + Nam Balb/c mice for 12 weeks. (4) WT C57BL/6J, WT CKD + HPD, IRF1^+/−^, IRF1^+/−^ CKD + HPD mice for 12 weeks. Male C57BL/6J mice of 8-week-old without surgery were divided into the following groups. (1) WT and *kl/kl* mice. (2) WT and IRF1^−/−^ mice. (3) WT and IRF1^−/−^ mice fed with or without HPD for 12 weeks. (4) Control and adenine-induced CKD mice (C57BL/6J mice) fed with a normal diet containing 0.25% adenine (Sigma) or not for 8 weeks. All animals were housed four to five per cage under a 12:12 h light:dark cycle at 25 °C, with a humidity of 40–70%.

High-resolution echocardiography was performed under anesthesia, and left ventricular internal diameter at diastole (LVIDd), left ventricular posterior wall thickness at diastole (LVPWd), EF% and short-axis M-mode views were recorded by the Vevo 770 Echocardiography Imaging System (VisualSonics, Toronto, ON, Canada). Mice were euthanized by carbon dioxide inhalation at appointed times, and the heart weight (HW), body weight (BW), and tibial length (TL) were recorded. Serum phosphate, BUN, and Scr were measured. The hearts were harvested for three parts: (1) perfused with 4% paraformaldehyde for hematoxylin-eosin (HE) and Wheat Germ Agglutinin (WGA) staining; (2) fixed in 2.5% glutaraldehyde at 4 °C for TEM observation; (3) froze in liquid nitrogen for qPCR and western blot analysis. All animal procedures were approved by the Animal Care and Use Committee of the Army Medical University, and were performed in accordance with the guidelines established by the Committee.

### Enzyme-linked immunosorbent assay

Enzyme-linked immunosorbent assay (ELISA) was performed using ELISA kits according to the manufacturer’s instructions. Mouse ELISA kits detecting serum intact FGF23, C­terminal FGF23 and PTH were bought from Immutopics (San Clemente, CA, USA). Mouse VitD ELISA kit was obtained from AbebioSience (Wuhan, China). Mouse klotho ELISA kit was acquired from Cusabio (Cologne, Germany).

### Cell culture

Rat embryonic cardiomyoblast cell line H9c2 was obtained from American Type Culture Collection (Manassas, VA, USA). Primary neonatal rat ventricular myocytes (NRVMs) were isolated as we and others previously described^11,48^. Briefly, the left ventricles of 1-day-old Sprague-Dawley rats (Beijing Huafukang Bioscience) were collected and digested with trypsin (Thermo Scientific, Waltham, MA, USA) at room temperature, and then incubated with 0.1% collagenase II (Solarbio Life Sciences, Beijing, China) for 30 min at 37 °C. Cells were filtered through a cell strainer (70 μm, BD Falcon, Lincoln Park, NJ, USA), and centrifuged at 1500 rpm for 10 min. Then, the cells were resuspended in Dulbecco’s Modified Eagle’s Medium (DMEM, Gibco, Grand Island, NY, USA) supplemented with 15% fetal bovine serum (FBS, Gibco) for 1 h, and in DMEM supplemented with 10 FBS and 0.1 mM bromodeoxyuridine (BrdU) for 48 h. Finally, both NRCMs and H9c2 cells were cultured in DMEM supplemented with 10% FBS and 1% (v/v) penicillin/streptomycin (Beyotime, Shanghai, China) at 37 °C and 5% CO_2_.

### Inorganic phosphate measurement

Intracellular inorganic phosphate in the supernatant of cell lysate was measured using the molybdate blue method with an inorganic phosphate assay kit (Jiancheng Bioengineering Institute, Nanjing, China) according to the manufacturer’s instructions. The corresponding protein concentration was determined using the BCA kit (Beyotime). The phosphate level of each sample was normalized against the protein content, and then compared with the control group for a relative intracellular phosphate level.

### Reverse transcription and qPCR

All tissue samples were collected on ice and flash-frozen using liquid nitrogen immediately. Samples were kept in liquid nitrogen for long-term storage, and were reserved in −80 °C refrigerator for short-term use. After isolation using Trizol (Invitrogen, Carlsbad, CA, USA) method, RNA was kept in 0.1% diethyl pyrocarbonate (DEPC) water, and the OD_260_/OD_280_ of RNA was detected using a NanoDrop 2000 spectrophotometer (Thermo Scientific). Only when the OD_260_/OD_280_ of RNA was between 1.8 and 2.0 did the RNA was used for subsequent reverse transcription using a reverse transcription kit (Promega, Madison, WI, USA). Real-time PCR was performed with SYBR Green qPCR kit (Takara, Dalian, China). The primers for qPCR are listed in Supplementary Tables 3 and 4.

### Western blot

Total protein was extracted with cell lysis buffer (Beyotime) supplemented with protease and phosphatase inhibitor cocktail (Roche Diagnostics GmbH, Mannheim, Germany), and the concentration was measured with BCA kit (Beyotime). The proteins were separated with 12% SDS-PAGE and transferred to PVDF membranes (ThermoScientific). The membranes were separately incubated with primary antibodies overnight at 4 °C, and anti-rabbit (A7016) or anti-mouse (A0216) HRP-conjugated secondary antibodies (Beyotime) for 1 h at room temperature^49^. Subsequently, the membranes were washed and the signals were visualized with Clarity ECL Substrate (Bio-Rad, Hercules, CA, USA). Gray-scale results were normalized against β-actin for semiquantitative analysis. The antibodies against IRF1 (sc-514544x), PPARα (sc-130640), and β-actin (sc-47778) were bought from Santa Cruz Biotechnology (Dallas, TX, USA). The antibodies against PGC1α (ab54481), Pit1 (ab10545), Pit2 (ab191182), phosphorylated FGFR1 (p-FGFR1, ab59194), total FGFR1 (t-FGFR1, ab824), phosphorylated FGFR4 (p-FGFR4, ab192589), total FGFR4 (t-FGFR4, ab41948), NRF1 (ab175932), and TFAM (ab131607) were obtained from Abcam Biotechnology (Cambridge, MA, USA). All of the antibodies were diluted 1:1000 for western blot. Uncropped and unprocessed scans of all blots are provided in Supplementary Figs. 17–21 in the Supplementary Information.

### ATP measurement

After cell lysis and centrifugation, the supernatant was determined using an ATP Determination Kit from Invitrogen company (A22066) according to the manufacturer’s instructions. The cellular ATP level was calculated according to the standard curve using an ATP reference standard. The corresponding protein concentration was tested using a BCA kit (Beyotime). ATP level (nmol) was normalized to total protein content (mg). Finally, each sample was compared with the control group for a relative ATP level.

### ROS detection

After HP treatment, H9c2 cells were washed with serum-free DMEM and incubated with CM-H2DCF-DA at 37 °C for 20 min, and analyzed using Accuri C6 flow cytometry (BD Biosciences, San Jose, CA, USA). A figure exemplifying the gating strategy is provided in Supplementary Fig. 22.

### Mitochondrial DNA analysis

Total DNA, including chromosomal (B2M) and mitochondrial (D-loop) DNA, was extracted from H9c2 cells using a DNA extraction kit (Takara) following the manufacturer’s instructions. Real-time PCR was carried out using an SYBR Green qPCR kit (Takara). Chromosomal DNA served as an internal control. The primers are shown in Supplementary Table 5.

### Mitochondrial membrane potential

H9c2 cells were treated with control or HP for 24 h. The cells were incubated with JC-1 at 37 °C for 20 min, washed twice with JC-1 buffer, and placed in the culture medium on ice. Cells were imaged using a confocal fluorescence microscope (Leica, Mannheim, Germany) or harvested for flow cytometry analysis (BD Biosciences).

### Transmission electron microscopy

Heart tissues of mice were harvested and fixed in 2.5% glutaraldehyde overnight at 4 °C and post fixed with 2% osmium tetroxide for 1 h at 37 °C. The samples were gradiently dehydrated in acetone (50%, 70%, 90%, and 100% separately) and embedded in epoxy resins. The ultrathin section was stained with uranyl acetate/lead citrate for visualization using a TEM (JEM-1400PLUS, Japan)^49^.

### Mitochondrial oxygen consumption rate analysis

OCR was assessed using the Seahorse XF96 Extracellular Flux Analyzer (Agilent Technologies). Cells were seeded quadruplicately in cell culture microplates (Agilent Technologies, Santa Clara, CA, USA) and treated with control or HP for 24 h. One hour prior to the assay, the culture medium was replaced by base medium (Agilent Technologies) supplemented with 1 mM sodium pyruvate, 2 mM glutamine, and 10 mM glucose (Agilent Technologies), and then cells were incubated at 37 °C without CO_2_. After three basal respiration measurements without additives, the ATP synthase inhibitor (1 μM oligomycin) was added, followed by subsequent mitochondrial uncoupler (1 μM FCCP), and complex I and III inhibitors (1 μM rotenone and 1 μM antimycin A), respectively. Three separate measurements were recorded after the reagents (all bought from Agilent Technologies) were injected.

### Laser scanning confocal microscopy

After treatment, cardiac cells were fixed with 4% paraformaldehyde for 20 min and stained with α-actinin (sc-17829, Santa Cruz Biotechnology, diluted 1:50) at 4 °C overnight. After washed with PBS, the cells were incubated with FITC-conjugated goat anti-mouse antibody (A0568, Beyotime, diluted 1:300) in the dark. Heart tissues were stained with WGA Alexa Fluor™ 555 Conjugate (500 μg/ml, W32464, Thermo Scientific) at room temperature for 10 min and washed with PBS four times. Cells and tissue sections were imaged using a confocal fluorescence microscope (Leica).

### Microarray analysis

Total RNA was extracted from the heart samples of sham and CKD mice for microarray analysis using Affymetrix Clariom S array (Affymetrix, Santa Clara, CA, USA), robust multiaverage, GO and KEGG pathway enrichment. Fold change >1.5 and *P* < 0.05 was defined as differentially expressed.

### Construction of overexpression plasmids

Full-length cDNA sequence of rat IRF1, PGC1α, NRF1, and TFAM was obtained from Pubmed. The corresponding primers, restriction enzymes and vectors are listed in Supplementary Table 6. PCR was performed to obtain the target gene fragments. After digestion, recombination, transformation, culture of the bacteria with recombinant plasmids, and further culture of the positive monoclones, the bacteria were identified by Beijing Genomics Institute (Beijing, China).

### Overexpression or silence of target genes

H9c2 cells were separately transfected with siRNAs (IRF1, Pit1, and Pit2), or overexpression plasmids (IRF1, PGC1α, NRF1, and TFAM) using Lipofectamine 2000 (Invitrogen) in OptiMEM (Hyclone, Logan, Utah, USA), according to the manufacturer’s protocol. Then, cells were treated with HP for another 24 h, and were harvested for the subsequent experiments. Scramble siRNA and siRNAs targeted to IRF1, Pit1, and Pit2 (listed in Supplementary Table 7) were synthesized by Sangon (Shanghai, China).

### Construction of reporter plasmids and point mutation

Putative IRF1 binding sites in the PGC1α promoter region are listed in Supplementary Table 1. Various lengths of the rat PGC1α promoter region were amplified by PCR using the genomic DNA of H9c2 cells as a template. The corresponding primers are listed in Supplementary Table 8. The fragments including PGC1α-1703 (−1703 to +188), PGC1α-1524 (−1524 to +188), PGC1α-985 (−985 to +188), PGC1α-719 (−719 to +188), PGC1α-348 (−348 to +188), and PGC1α-183 (−183 to +188) were separately cloned into a pGL3-basic vector (Promega) after digestion with HindIII, and the recombinant reporter plasmids were separately named as pGL3-PGC1α-P6, pGL3-PGC1α-P5, pGL3-PGC1α-P4, pGL3-PGC1α-P3, pGL3-PGC1α-P2, and pGL3-PGC1α-P1. pGL3-PGC1α-M3, containing point mutations in the IRF1 binding element (AATAGGGAGGAGAGGGAAGGA, the mutated bases are underlined), was generated with MutanBEST kit (Takara) using pGL3-PGC1α-P3 (−719 to +188) as a template. Negative control mutation plasmid pGL3-PGC1α-M4, containing point mutations in the IRF1 binding element (ATATAAGAAGGGAGGGGGGGG, the mutated bases are underlined), was also generated, using pGL3-PGC1α-P4 (−985 to +188) as a template.

### Dual-luciferase reporter assay

The recombinant reporter plasmids were co-transfected with pRL-TK vector (Promega) into H9c2 cells using Lipofectamine 2000 (Invitrogen) in OptiMEM (Hyclone). Then, the cells were treated with control or HP for another 24 h. Luciferase activity was detected using the Dual-luciferase reporter assay system (Promega). The firefly luciferase activity was normalized against Renilla activity. All transfection experiments were performed three times in triplicate.

### Chromatin immunoprecipitation

H9c2 cells were treated with control or HP for 24 h, and then fixed with 1% formaldehyde for 10 min. The cells were lyzed in SDS lysis buffer. The chromatin was sonicated to shear DNA to an average length between 200 to 1000 bp, and immunoprecipitated with 2 μg antibody against IRF1 (sc-514544x, Santa Cruz Biotechnology), taking IgG as a negative control. The precipitated DNA was amplified by PCR and qPCR with the primers (−709 to −511) that cover the IRF1 binding sites (−632 to −612). Primers (−1711 to −1598) without IRF1 binding sites served as a negative control, while the total DNA (Input) served as a positive control. For acetylation detection, the sonicated DNA was immunoprecipitated with 2 μg antibody against H3 (Lys9) (#9671) or H4 (Lys12) (#13944) (Cell Signaling Technology, Danvers, MA, USA), taking IgG as a negative control. The primers for ChIP are listed in Supplementary Table 9.

### Methylation-specific PCR

Genomic DNA of H9c2 cells was modified with bisulfate using EZ DNA Methylation Gold Kit (Zymo Research, Irvine, CA) according to the manufacturer’s instructions. The primers that recognize the methylated and unmethylated CpG island sites in the IRF1 promoter region were designed by MethPrimer software^50^ (http://www.urogene.org/methprimer/) and listed in Supplementary Table 10. The PCR products of genomic DNA without bisulfate modification served as a positive control (Input).

### Statistical analysis

All continuous characteristics were plotted by bar charts (means and standard deviations). Comparisons of indicators that follow normal distribution between two groups were analyzed by two independent sample *t*-test, and that among multiple groups were tested by one-way analysis of variance (ANOVA). Considering treatment time factors, comparisons of indicators between treatment groups were examined using analysis of variance of factorial design. In the human study, continuous characteristics that obey non-normal distribution were drawn by box plots (medians and interquartiles). Spearman rank correlation was used to analyze the correlation between phosphate levels and eGFR, and partial correlation analyses were performed to examine the associations of EF and LVMI with phosphate levels after controlling eGFR. Kruskal–Wallis *H* test was used to compare the indicators among three tertiles of the serum phosphate levels. Statistical analyses were performed using Graphpad Prism 8.0 and SPSS 21.0, and all tests were two-tailed with a significant level set at *P* < 0.05.

### Reporting summary

Further information on research design is available in the Nature Research Reporting Summary linked to this article.

## Supplementary information

Supplementary Information

Reporting Summary

## Data Availability

All raw data of microarray analyses have been submitted to Gene Expression Omnibus (GEO) database (accession no. GSE143031). Source data are provided with this paper. Other data that support the findings of this study are available within the article and Supplementary files. Source data are provided with this paper.

## Source data

Source Data
