# Supplementary material for: IRF1-mediated downregulation of PGC1α contributes to cardiorenal syndrome type 4
Source: Nat Commun. 2020 Sep 16;11:4664. doi: 10.1038/s41467-020-18519-0 (PMC7494935; doi:10.1038/s41467-020-18519-0)
Supplement: Supplementary file 1 — Supplementary Information [file 41467_2020_18519_MOESM1_ESM.pdf]

IRF1-mediated downregulation of PGC1α contributes to cardiorenal syndrome type 4

Huang *et al*.

Supplementary Figure 1

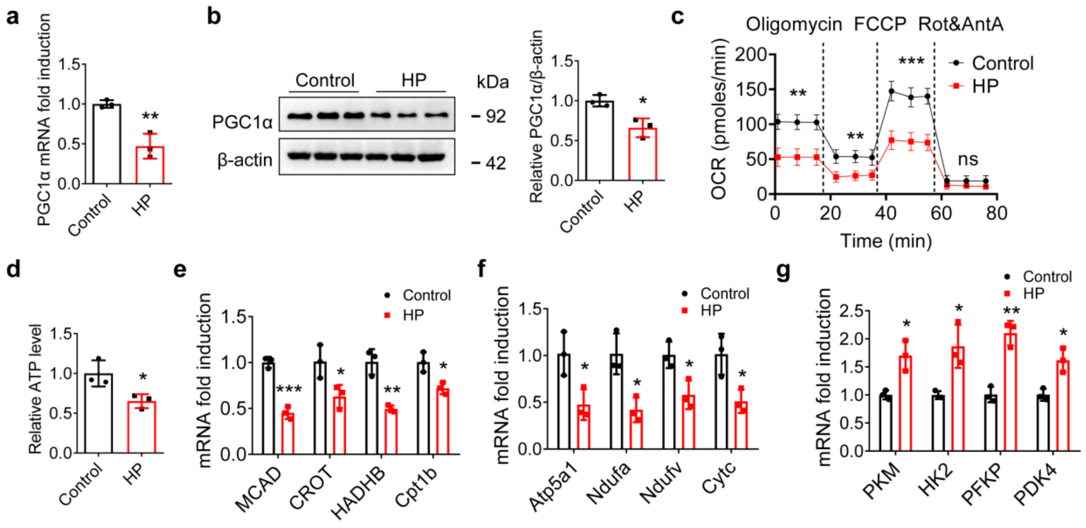

Supplementary Figure 1. HP induces PGC1α downregulation and energy metabolic remodeling in primary neonatal rat ventricular myocytes (NRVMs).

a, **b** Primary neonatal rat ventricular myocytes (NRVMs) were treated with control or HP for 24 h. Then, cells were collected for qPCR and Western blot analysis of PGC1α expression. **c** Oxygen consumption rate (OCR) of cells in (**a**) was determined using a Mito Stress test kit (n = 4). **d** Relative ATP level of cells in (**a**) was measured using an ATP determination kit and a BCA kit. **e-g** qPCR analysis of the mRNA expression of FAO-related genes (**e**), OXPHO-related genes (**f**) and glycolysis-related genes (**g**) of cells in (**a**). β-actin served as an internal control. Data are shown as mean ± SD and were analyzed by two-tailed unpaired *t* test (**a-g**). n = 3 except (**c**) (n = 4) biologically independent experiments. * $P < 0.05$, ** $P < 0.01$, *** $P < 0.001$. ns: no significance. Source data are provided as a Source Data file.

Supplementary Figure 2

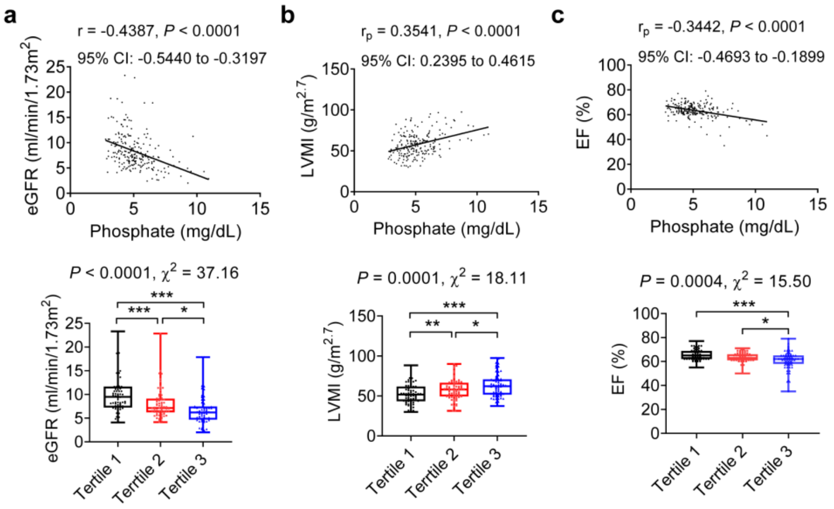

Supplementary Figure 2. High phosphate correlates with cardiac hypertrophy and heart failure in CKD patients.

a-c Serum phosphate level was negatively associated with estimated glomerular filtration rate (eGFR) (**a**) and left ventricular ejection fraction (EF%) (**c**), and positively correlated with left ventricular mass index (LVMI, calculated by indexing left ventricular mass to height$^{2.7}$) (**b**) in 213 predialysis CKD patients. The association of eGFR with phosphate levels was analyzed by Spearman rank correlation, and the associations of LVMI and EF% with phosphate levels were performed using partial correlation analysis to control the confounding of eGFR (upper panel). The CKD patients were divided into three groups according to tertiles of serum phosphate level: tertile 1 (2.787 - 4.398 mg/dL) (n = 72), tertile 2 (4.491 - 5.513 mg/dL) (n = 70) and tertile 3 (5.544 - 10.932 mg/dL) (n = 71). eGFR, LVMI and EF% were shown in box plots with all individual points, where the middle line is the median (**a-c**, lower panel). The lower and upper ends of the box indicate the first and third quartiles, respectively. The upper and lower whiskers of the box plot indicate the maximum and the minimum individual point, respectively. The data does not follow a normal distribution. The comparisons among

multiple groups were analyzed by Kruskal-Wallis *H* test (lower panel) using SPSS 21.0. A two-tailed $P < 0.05$ was considered statistically significant. * $P < 0.05$, ** $P < 0.01$, *** $P < 0.001$. Source data are provided as a Source Data file.

Supplementary Figure 3

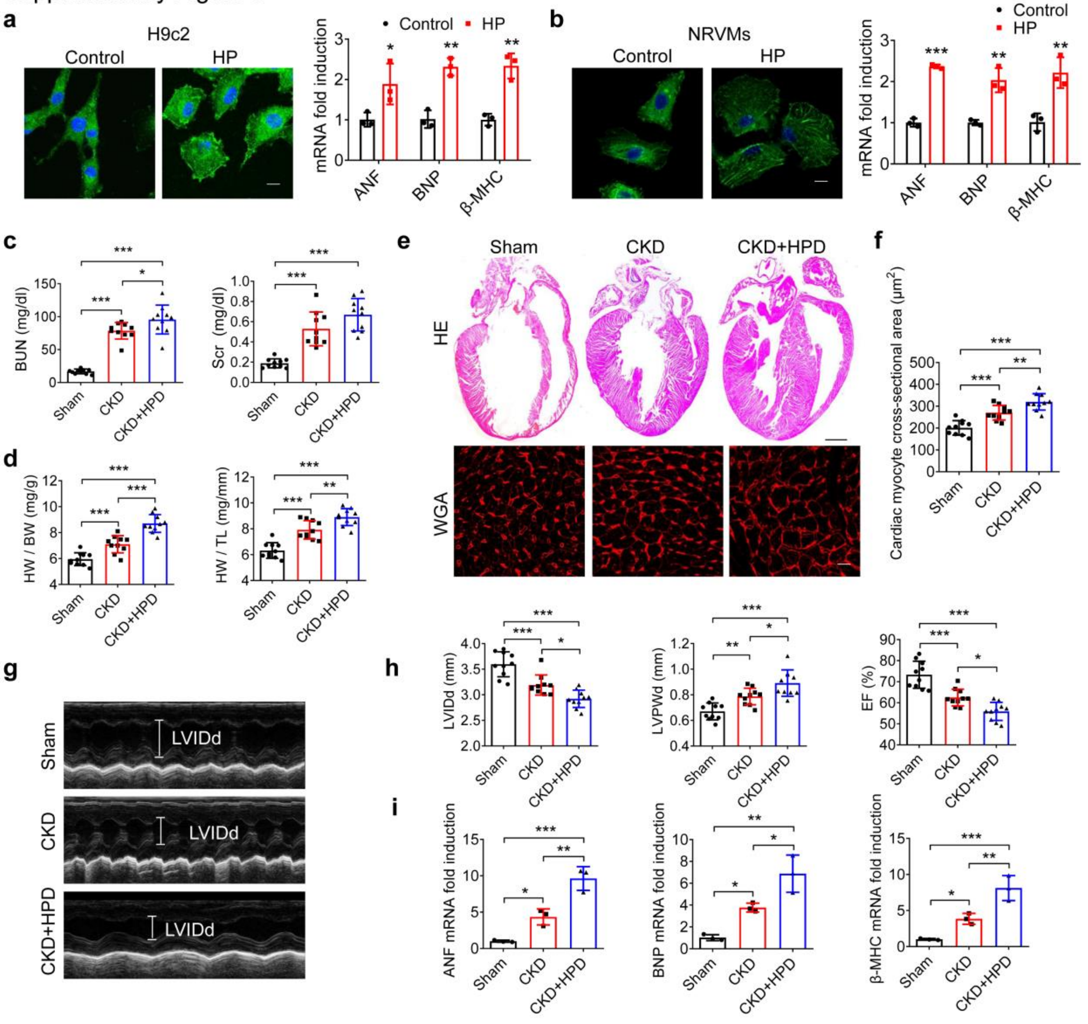

Supplementary Figure 3. PGC1α inhibition and mitochondrial energy metabolism remodeling are induced in HP-treated CKD mice.

a, **b** H9c2 cells (**a**) and NRVMs (**b**) were treated with control or HP for 24 h. The cell size was determined by immunofluorescence staining using α-actinin antibody. Scale bar, 20 μm. The expression of hypertrophic genes (ANF, BNP and β-MHC) was detected using qPCR. **c** The blood urea nitrogen (BUN) and serum creatinine (Scr) of sham, CKD and high phosphate diet (HPD)-fed CKD mice for 12 weeks. **d** The ratio of heart weight (HW) to body weight (BW) and HW to tibial length (TL) of mice in (**c**). **e** Representative gross pathology of heart sections (HE staining, upper panel. Scale bar 1 mm) and WGA staining of left ventricular of

heart sections (lower panel. Scale bar 10 μm) from mice in (**c**). **f** Cross-sectional surface area of individual cardiac myocytes from mice in (**e**, lower panel). **g**, **h** The LVIDd, LVPWd and EF% of mice in (**c**) were determined by echocardiography. **i** qPCR analysis of relative mRNA expression of hypertrophic genes (ANF, BNP and β-MHC) in heart lysates from mice in (**c**). β-actin served as an internal control. Data are shown as mean ± SD and were analyzed by two-tailed unpaired *t* test (**a**, **b**) or one-way ANOVA (**c-i**). n = 3 (**a**, **b**, **i**), n = 10 mice per group (**c-h**). * $P < 0.05$, ** $P < 0.01$, *** $P < 0.001$. Source data are provided as a Source Data file.

Supplementary Figure 4

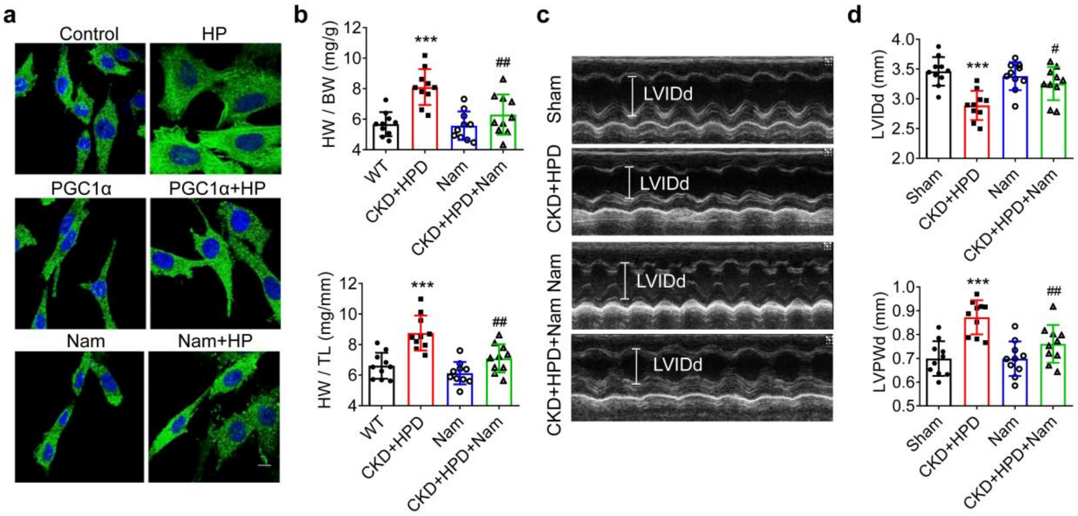

Supplementary Figure 4. Restoration of PGC1α rescues HP-induced cardiac hypertrophy.

a H9c2 cells were transfected with PGC1α overexpression plasmids or pretreated with Nam, and then treated with control or HP for 24 h. Cardiac shape was detected using laser scanning confocal microscopy. Scale bar, 20 μm. **b** The ratio of HW to BW and HW to TL of sham mice, HPD-fed CKD mice, Nam-treated mice and HPD-fed CKD mice intraperitoneally injected with Nam for 12 weeks. **c**, **d** Echocardiography detection of LVIDd and LVPWd of mice in (**b**). Data are shown as mean ± SD and were analyzed by one-way ANOVA. n = 10 mice per group. *** $P < 0.001$ versus sham. $^{\#}$ $P < 0.05$, $^{\#\#}$ $P < 0.01$ versus CKD+HPD. Source data are provided as a Source Data file.

Supplementary Figure 5

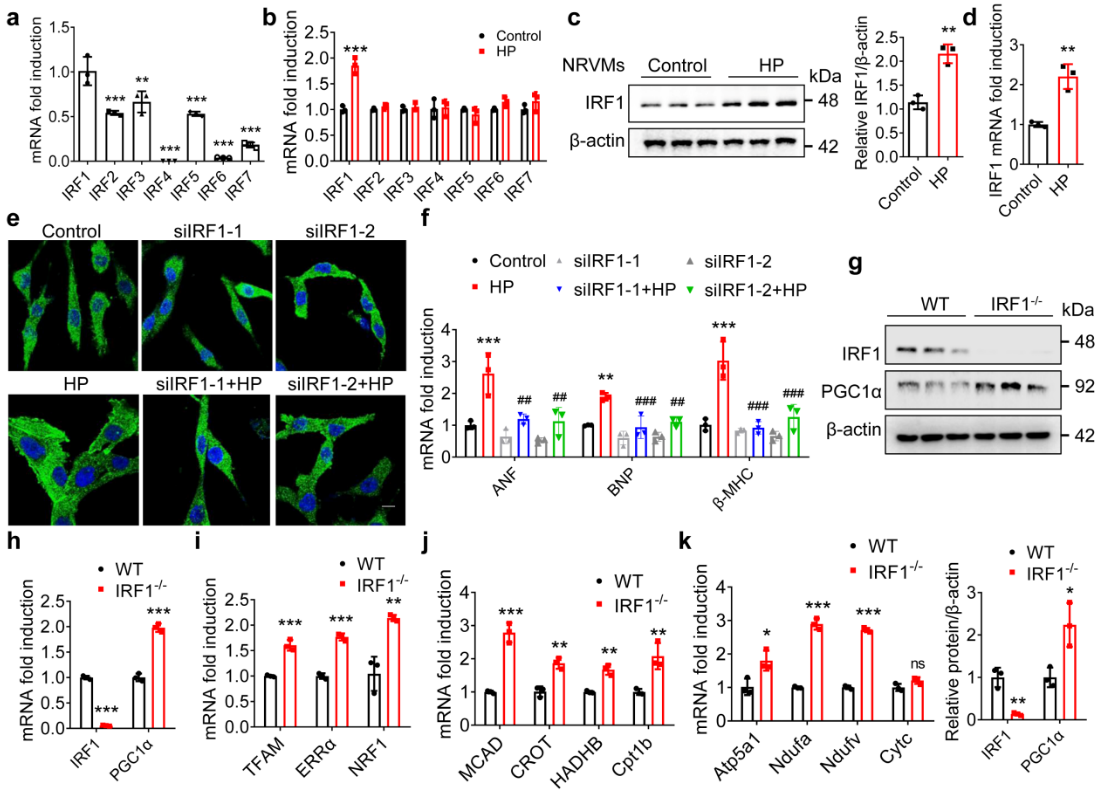

Supplementary Figure 5. HP-induced upregulation of IRF1 promotes cardiac hypertrophy.

a qPCR analysis of IRF family members in H9c2 cells. **b** qPCR analysis of IRF family members in H9c2 cells treated with control or HP for 24 h. **c**, **d** Western blot and qPCR analysis of IRF1 expression in neonatal rat ventricular myocytes (NRVMs) treated with control or HP for 24 h. **e**, **f** H9c2 cells were transfected with two pairs of siRNA against IRF1, and then treated with control or HP for another 24 h to detect cardiac shape using laser scanning confocal microscopy (**e**) and the expression of hypertrophic genes using qPCR (**f**). Scale bar, 20 μm. **g**, **h** Western blot and qPCR analysis of IRF1 and PGC1α expression in wild type (WT) and IRF1 knockout (IRF1$^{-/-}$) mice. **i-k** qPCR analysis of the expression of PGC1α target genes (**i**), FAO genes (**j**) and OXPHO genes (**k**) in heart lysates from WT and IRF1$^{-/-}$ mice. Data are shown as mean ± SD and were analyzed by one-way ANOVA (**a**, **f**) or

two-tailed unpaired *t* test (**b-d**, **g-k**). n = 3 biologically independent experiments (**a-k**). * P < 0.05, ** P < 0.01, *** P < 0.001 versus control. $^{\#\#}$ P < 0.01, $^{\#\#\#}$ P < 0.001 versus HP. ns: no significance. Source data are provided as a Source Data file.

Supplementary Figure 6

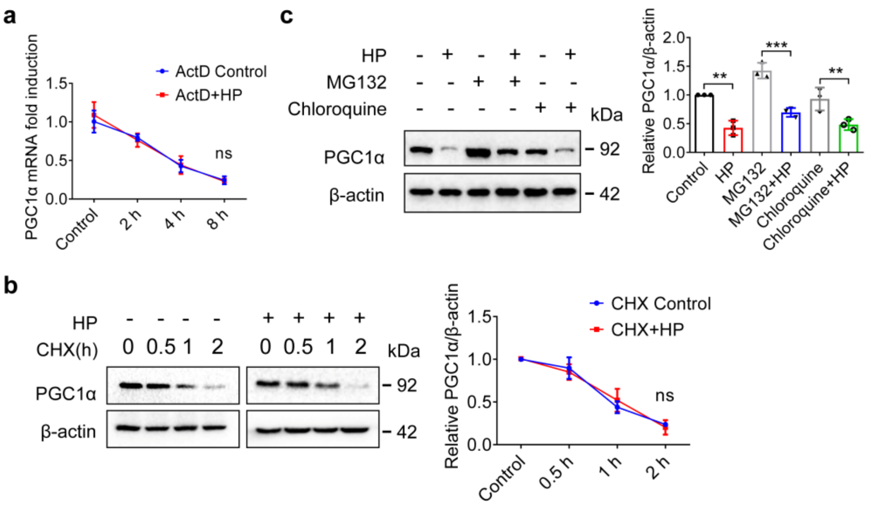

Supplementary Figure 6. HP can regulate neither the mRNA stability nor the protein degradation of PGC1α.

a Cardiac cells were treated with a transcriptional inhibitor actinomycin D (ActD, 0.5 μg/ml) for various time in the absence or presence of HP. PGC1α mRNA expression was determined using qPCR. **b** Cells were treated with a translational inhibitor cycloheximide (CHX, 10 μM) time-dependently in the absence or presence of HP. PGC1α protein expression was detected using Western blot. **c** Cells were treated with a proteasomal inhibitor MG132 (10 μM) or a lysosomal inhibitor chloroquine (20 μmol/L) in combination with or without HP. PGC1α protein expression was detected using Western blot (representative images). β-actin served as an internal control. Data are shown as mean ± SD and were analyzed by one-way ANOVA (**c**) or two-way ANOVA (**a**, **b**). n = 3 biologically independent experiments (**a-c**). ** $P < 0.01$, *** $P < 0.001$. ns: no significance. Source data are provided as a Source Data file.

Supplementary Figure 7

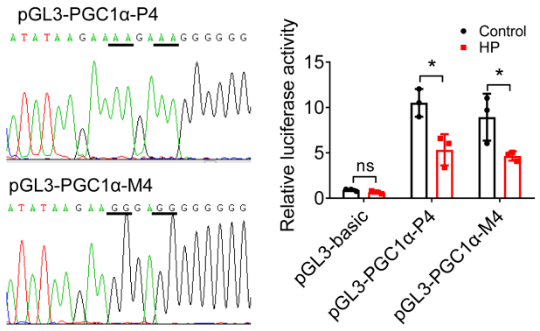

Supplementary Figure 7. Mutation of PGC1α promoter region P4 has no effect on HP-inhibited PGC1α promoter activity.

H9c2 cells were co-transfected with pRL-TK vector and pGL3-PGC1α-P4 or pGL3-PGC1α-M4 (containing the mutant bases of pGL3-PGC1α-P4 that were underlined), and treated with control or HP for 24 h. Cells were harvested for dual-luciferase reporter assay. The firefly luciferase activity was normalized against Renilla activity. pGL3-basic served as a control. Data are shown as mean ± SD and were analyzed by two-tailed unpaired *t* test. n = 3 biologically independent experiments. * $P < 0.05$. ns: no significance. Source data are provided as a Source Data file.

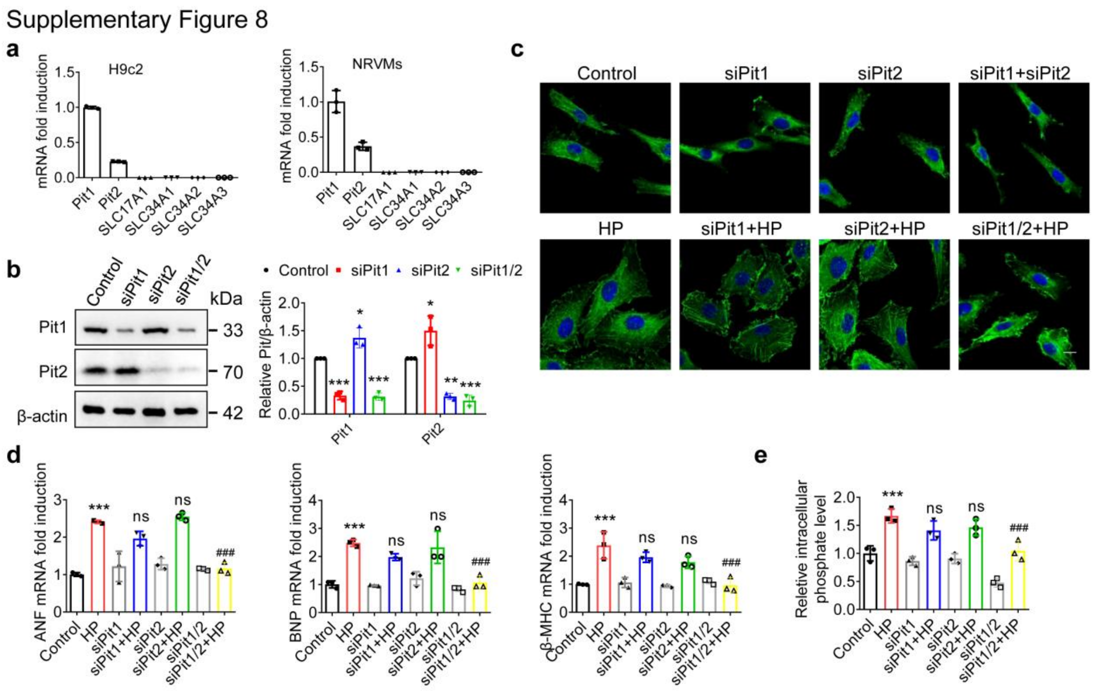

Supplementary Figure 8. Pit1 and Pit2 mediate HP-induced cardiac hypertrophy.

a qPCR analysis of the expression of phosphate transporters in both H9c2 cells and NRVMs. **b** Representative Western blot analysis of Pit1 and Pit2 expression in H9c2 cells transfected with siRNA against Pit1(siPit1), Pit2(siPit1) or both (siPit1/2). **c-e** H9c2 cells were transfected with control, siPit1, siPit2 or siPit1/2, and then treated with or without HP for another 24 h. The cell size was determined by immunofluorescence staining using α-actinin antibody (**c**). Scale bar, 20 μm. The expression of hypertrophic genes was detected using qPCR (**d**). Intracellular phosphate level was measured using a phosphate detection kit, and normalized against the protein content. Each sample was compared with the control group for a relative phosphate level (**e**). Data are shown as mean ± SD and were analyzed by one-way ANOVA. n = 3 biologically independent experiments (**a**, **b**, **d**, **e**). * $P < 0.05$, ** $P < 0.01$, *** $P < 0.001$ versus control. ### $P < 0.001$ versus HP. ns: no significance versus HP. Source data are provided as a Source Data file.

Supplementary Figure 9

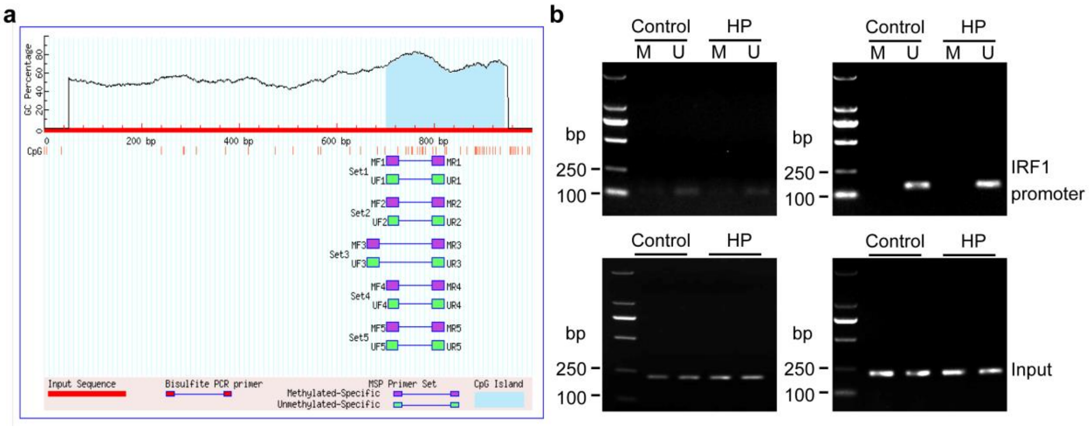

Supplementary Figure 9. HP cannot induce methylation of IRF1 promoter.

a CpG island analysis in the promoter region of *IRF1* gene was detected. **b** H9c2 cells were treated with control or HP for 24 h, and harvested for methylation specific PCR (MSP) detection using two different primers that recognize the methylated (M) and unmethylated (U) CpG island sites designed by MethPrimer software. The PCR products of genomic DNA without bisulfate modification served as a positive control (Input).

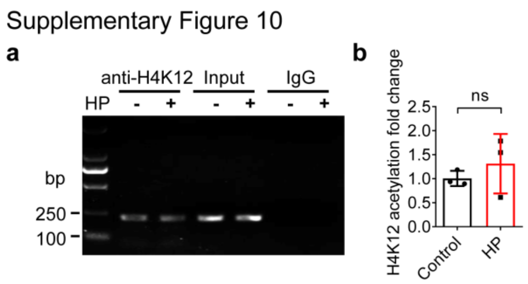

Supplementary Figure 10. HP cannot induce H4K12 acetylation in IRF1 promoter region.

a, **b** H9c2 cells were treated with control or HP for 24 h, and H4K12 acetylation was assayed using ChIP. DNA incubated with IgG served as a negative control. DNA incubated with no antibodies served as a positive control (Input). The binding DNA was detected using PCR (**a**) and qPCR (**b**). Data are shown as mean ± SD and were analyzed by two-tailed unpaired *t* test (**b**). n = 3 biologically independent experiments. ns: no significance. Source data are provided as a Source Data file.

Supplementary Figure 11

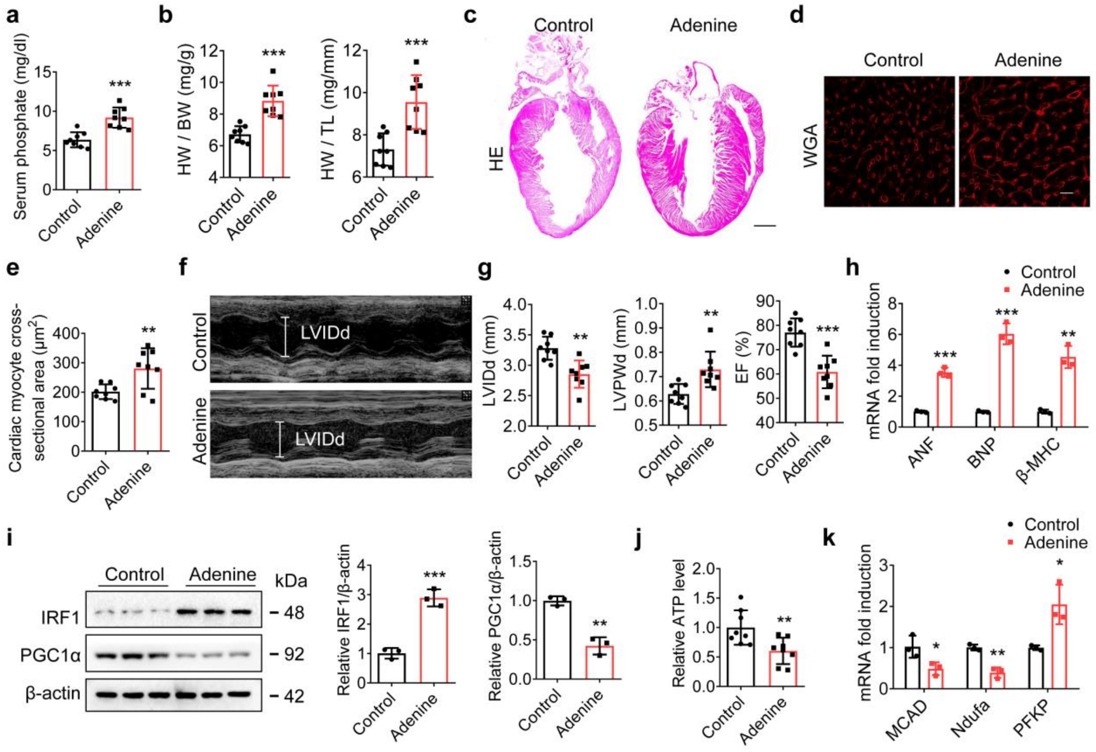

Supplementary Figure 11. Higher phosphate level and cardiac hypertrophy are observed in adenine-induced CKD model.

a Serum phosphate level of normal mice fed with control or adenine diet (0.25%) for 8 weeks. **b** The ratio of HW to BW and HW to TL of control and adenine mice. **c-e** Representative gross pathology of heart sections (**c**. HE staining, Scale bar 1 mm), WGA staining of left ventricular of heart sections (**d**. Scale bar 10 μm) and cross-sectional surface area of individual cardiac myocytes (**e**) from mice in (**a**). **f**, **g** Echocardiography detection of LVIDd, LVPWd and EF% of mice in (**a**). **h-k** qPCR analysis of hypertrophic genes expression (**h**), Western blot analysis of IRF1 and PGC1α expression (**i**), relative ATP level (**j**) and relative mRNA expression of metabolic genes (**k**) of heart lysates from the mice in (**a**).β-actin was taken as the loading control. Data are shown as mean ± SD and were analyzed by two-tailed unpaired *t* test (**a**, **b**, **e**, **g-k**). n = 8 mice per group (**a-g**, **j**). n = 3 biologically independent

experiments (**h**, **i**, **k**). * $P < 0.05$, ** $P < 0.01$, *** $P < 0.001$. Source data are provided as a Source Data file.

Supplementary Figure 12

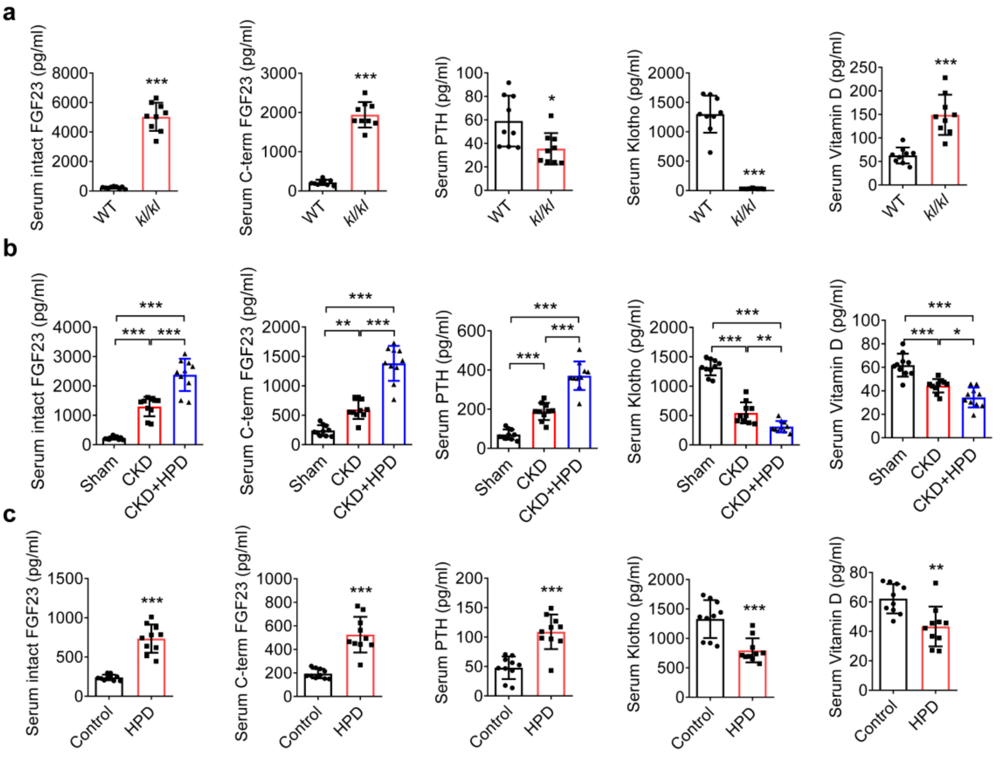

Supplementary Figure 12. Serological tests of FGF23, PTH, klotho and Vitamin D in wild type and *kl/kl* mice; sham, CKD mice fed with or without high phosphate diet (HPD); and normal mice fed with control or HPD.

a-c Serum levels of intact FGF23, C-terminal FGF23 (C-term FGF23), PTH, klotho and Vitamin D (VitD) were determined using the corresponding ELISA kits in wild type and *kl/kl* mice (**a**) (n = 9 mice per group); sham, CKD mice fed with or without high phosphate diet (HPD) (**b**) (n = 10 mice per group); and normal mice fed with control or HPD (**c**) (n = 10 mice per group). Data are shown as mean ± SD and were analyzed by two-tailed unpaired *t* test (**a**, **c**) or one-way ANOVA (**b**). * $P < 0.05$, ** $P < 0.01$, *** $P < 0.001$. Source data are provided as a Source Data file.

Supplementary Figure 13

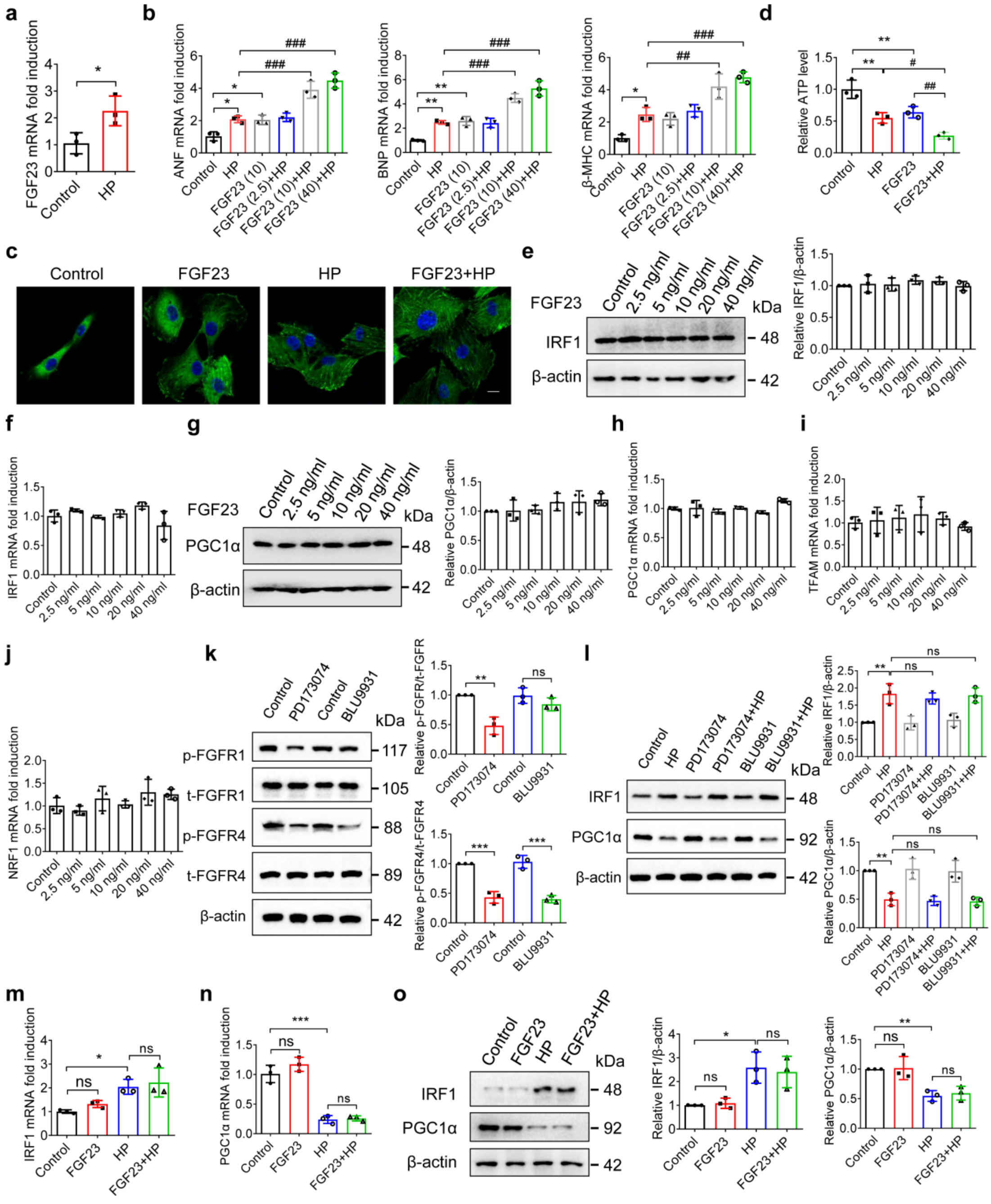

Supplementary Figure 13. HP-induced cardiac hypertrophy is independent of FGFR, and FGF23-enhanced cardiac hypertrophy doesn’t depend on IRF1-PGC1α axis, although HP and FGF23 exhibit additive effects.

a qPCR analysis of FGF23 mRNA expression in H9c2 cells treated with control or HP for 24 h. **b** qPCR analysis of the expression of hypertrophic genes in H9c2 cells treated with HP, FGF23 alone, or HP in combination with increasing doses of FGF23 for 24 h. **c**, **d** The

immunofluorescence stained with α-actinin (**c**) and relative ATP level (**d**) in H9c2 cells treated with FGF23 in the absence or presence of HP for 24 h. Scale bar, 20 μm. **e-h** qPCR and representative Western blot analysis of the expression of IRF1 and PGC1α in H9c2 cells treated with control or various doses of FGF23 for 24h. **i**, **j** qPCR analysis of the expression of PGC1α target genes (TFAM and NRF1) in cells in (**h**). **k** The phosphorylation of FGFR1 and FGFR4 were detected using Western blot in H9c2 cells treated with a Pan-FGFR inhibitor (PD173074) or a FGFR4 inhibitor (BLU9931) for 24 h (representative images). **l** Representative Western blot analysis of IRF1 and PGC1α expression in H9c2 cells treated with PD173074 or BLU9931 in the presence or absence of HP. **m-o** qPCR and representative Western blot analysis of IRF1 and PGC1α expression in cells in (**c**). Data are shown as mean ± SD and were analyzed by two-tailed unpaired t test (**a**, **k**) or one-way ANOVA (**b**, **d-j**, **l-o**). n = 3 biologically independent experiments (**a**, **b**, **d-o**). * $P < 0.05$, ** $P < 0.01$, *** $P < 0.001$ versus Control. ## $P < 0.01$, ### $P < 0.001$ versus HP. ns: no significance. Source data are provided as a Source Data file.

Supplementary Figure 14

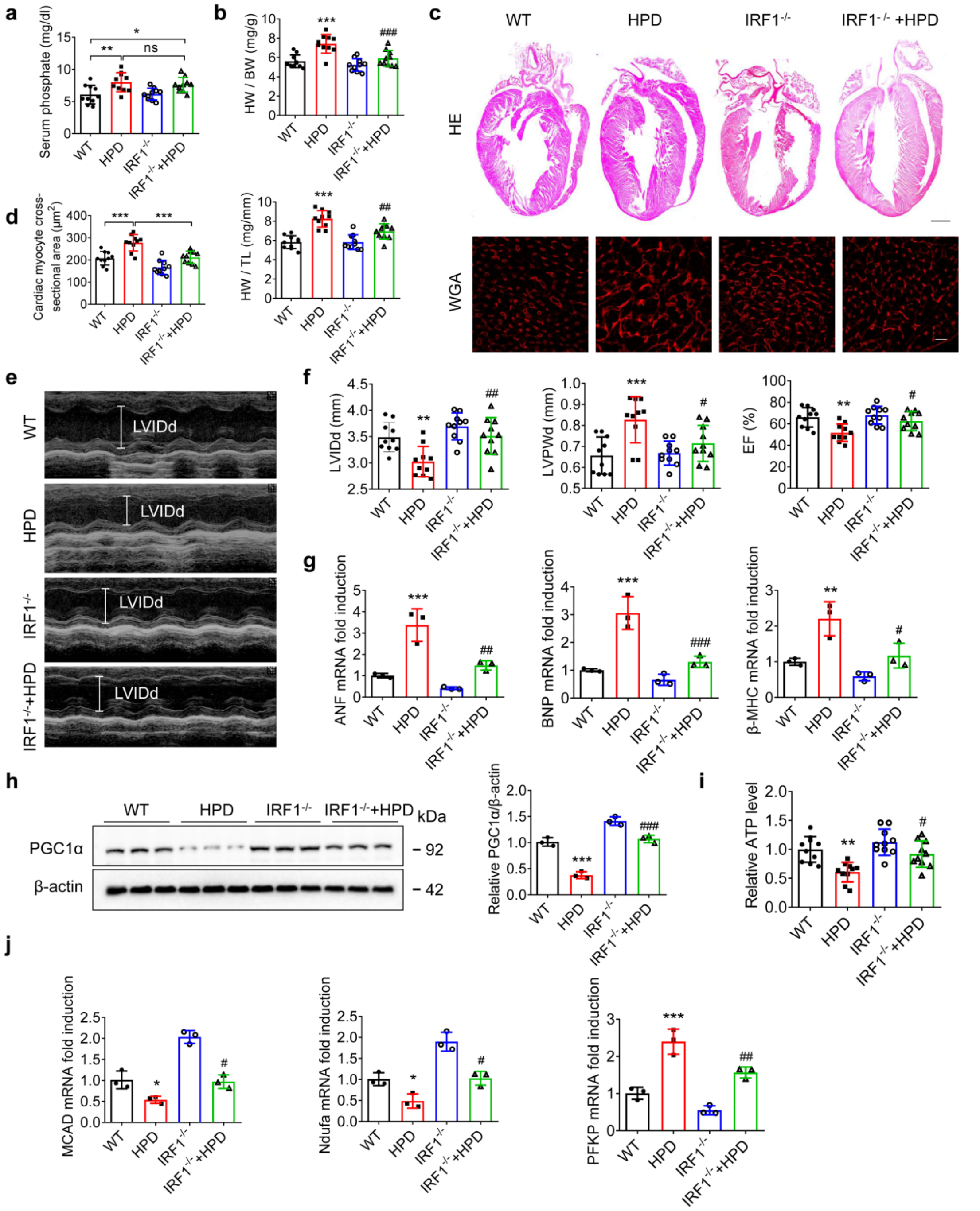

Supplementary Figure 14. HPD-induced cardiac hypertrophy and energy metabolism remodeling are significantly attenuated in IRF1 knockout mice.

a Serum phosphate level of wild type (WT) and IRF1 knockout (IRF1$^{-/-}$) mice fed with control or high phosphate diet (HPD) for 12 weeks. n = 10 mice per group. **b** The ratio of HW to BW and HW to TL of WT, HPD, IRF1$^{-/-}$ and IRF1$^{-/-}$ +HPD mice. **c** Representative gross

pathology of heart sections (HE staining, upper panel. Scale bar 1 mm) and WGA staining of left ventricular of heart sections (lower panel. Scale bar 10 μm) from mice in (**a**). **d** Cross-sectional surface area of individual cardiac myocytes from mice in (**c**, lower panel). **e**, **f** Echocardiography detection of LVIDd, LVPWd and EF% of mice in (**a**). **g**-**j** qPCR analysis of hypertrophic genes expression (**g**), Western blot analysis of PGC1α expression (**h**), relative ATP level (**i**) and relative mRNA expression of metabolic genes (**j**) of heart lysates from the mice in (**a**). β-actin was taken as the loading control. Data are shown as mean ± SD and were analyzed by one-way ANOVA. n = 10 mice per group (**a**-**f**, **i**). n = 3 biologically independent experiments (**g**, **h**, **j**). * $P < 0.05$, ** $P < 0.01$, *** $P < 0.001$ versus WT. $^{\#}$ $P < 0.05$, $^{\#}$ $P < 0.05$, $^{\#\#}$ $P < 0.01$, $^{\#\#\#}$ $P < 0.001$ versus HPD. Source data are provided as a Source Data file.

Supplementary Figure 15

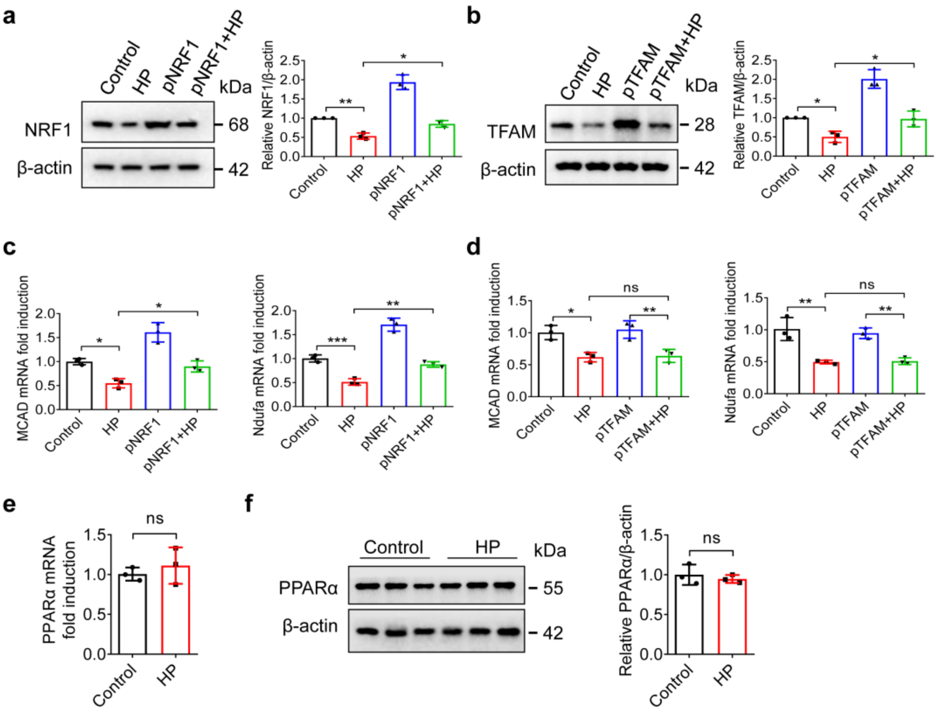

Supplementary Figure 15. HP cannot regulate PPARα expression, while HP-induced dysregulation of FAO and OXPHO-related genes was significantly attenuated by overexpression of NRF1, rather than TFAM.

a-d H9c2 cells were transfected with overexpression plasmids of NRF1 (pNRF1) or TFAM (pTFAM), and then treated with control or HP for another 24 h. Cells were harvested for Western blot analysis (representative images) of NRF1 or TFAM expression, and qPCR analysis of MCAD and Ndufa expression. **e-f** qPCR (**e**) and Western blot (**f**) analysis of PPARα expression in H9c2 cells treated with control or HP for 24 h. Data are shown as mean ± SD and were analyzed by one-way ANOVA (**a-d**) or two-tailed unpaired t test (**e**, **f**). n = 3 biologically independent experiments (**a-f**). * $P < 0.05$, ** $P < 0.01$, *** $P < 0.001$. ns: no significance. Source data are provided as a Source Data file.

Supplementary Figure 16

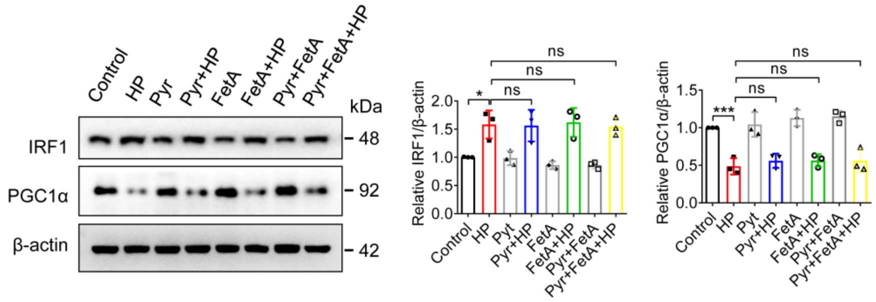

Supplementary Figure 16. Neither pyrophosphate nor fetuin A can regulate HP-induced IRF1 upregulation or PGC1α downregulation.

Representative Western blot analysis of IRF1 and PGC1α expression in H9c2 cells treated with control, pyrophosphate (Pyr, 10 uM), fetuin A (FetA, 5 μM) or both in the absence or presence of HP for 24 h. Data are shown as mean ± SD and were analyzed by one-way ANOVA. n = 3 biologically independent experiments. * $P < 0.05$, *** $P < 0.001$. ns: no significance. Source data are provided as a Source Data file.

Supplementary Figure 17. Uncropped gel images for Figure 1-5.

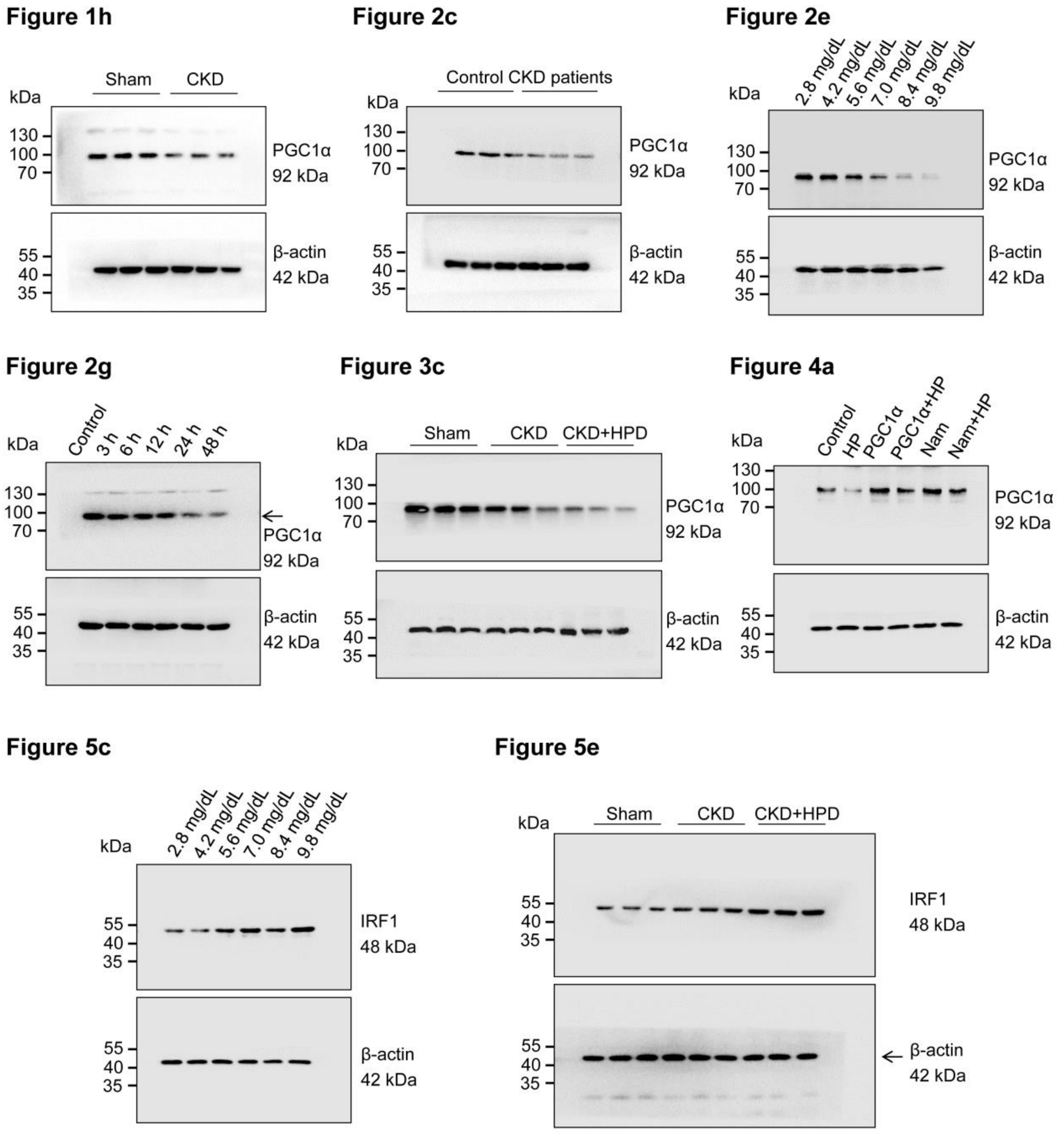

Supplementary Figure 18. Uncropped gel images for Figure 5-6.

Figure 5g

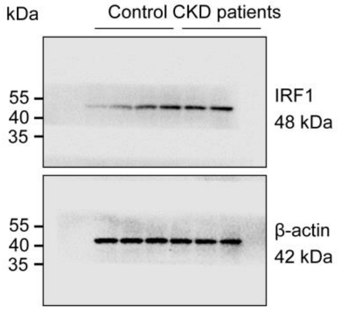

Figure 5h

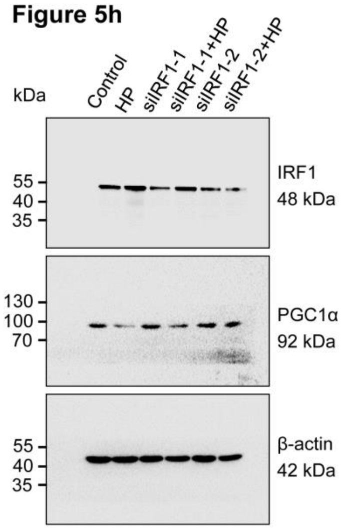

Figure 5n

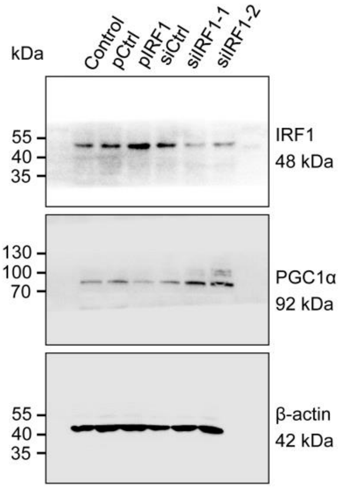

Figure 6c

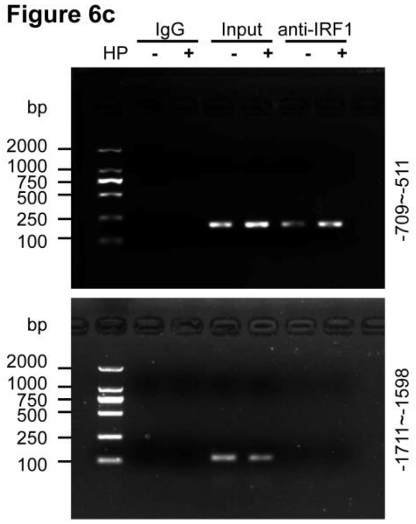

Figure 6e

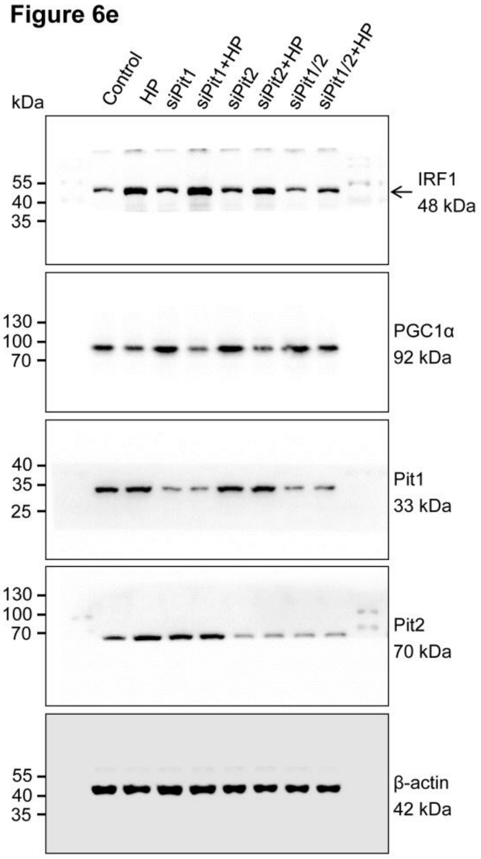

Figure 6g

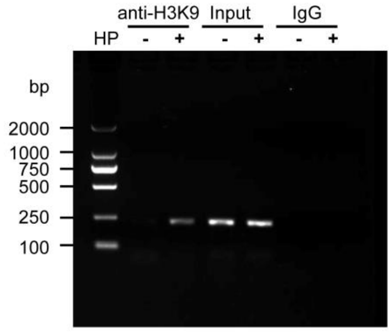

Supplementary Figure 19. Uncropped gel images for Figure 6-8, S1, S5 and S6.

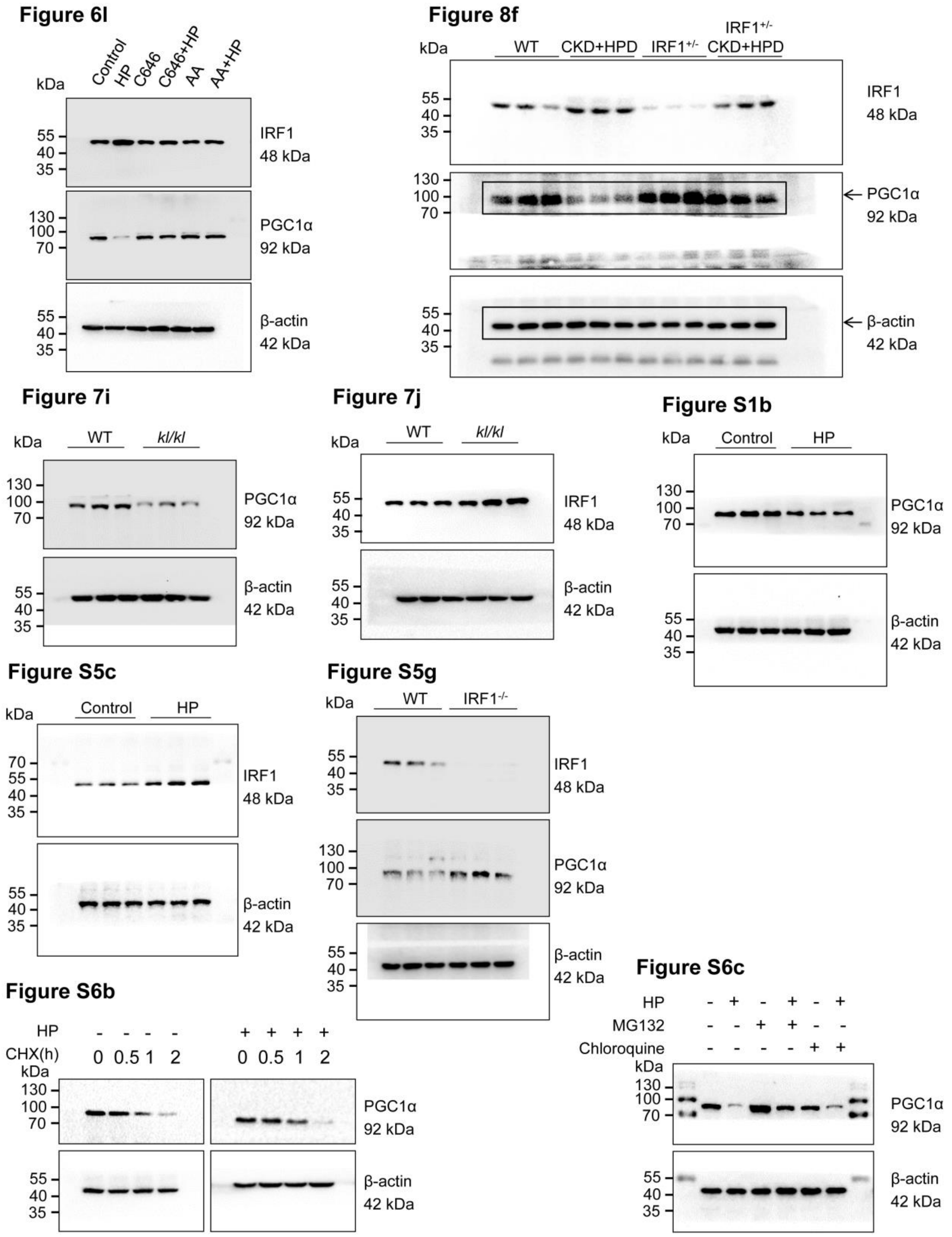

Supplementary Figure 20. Uncropped gel images for Figure S8-S11 and S13.

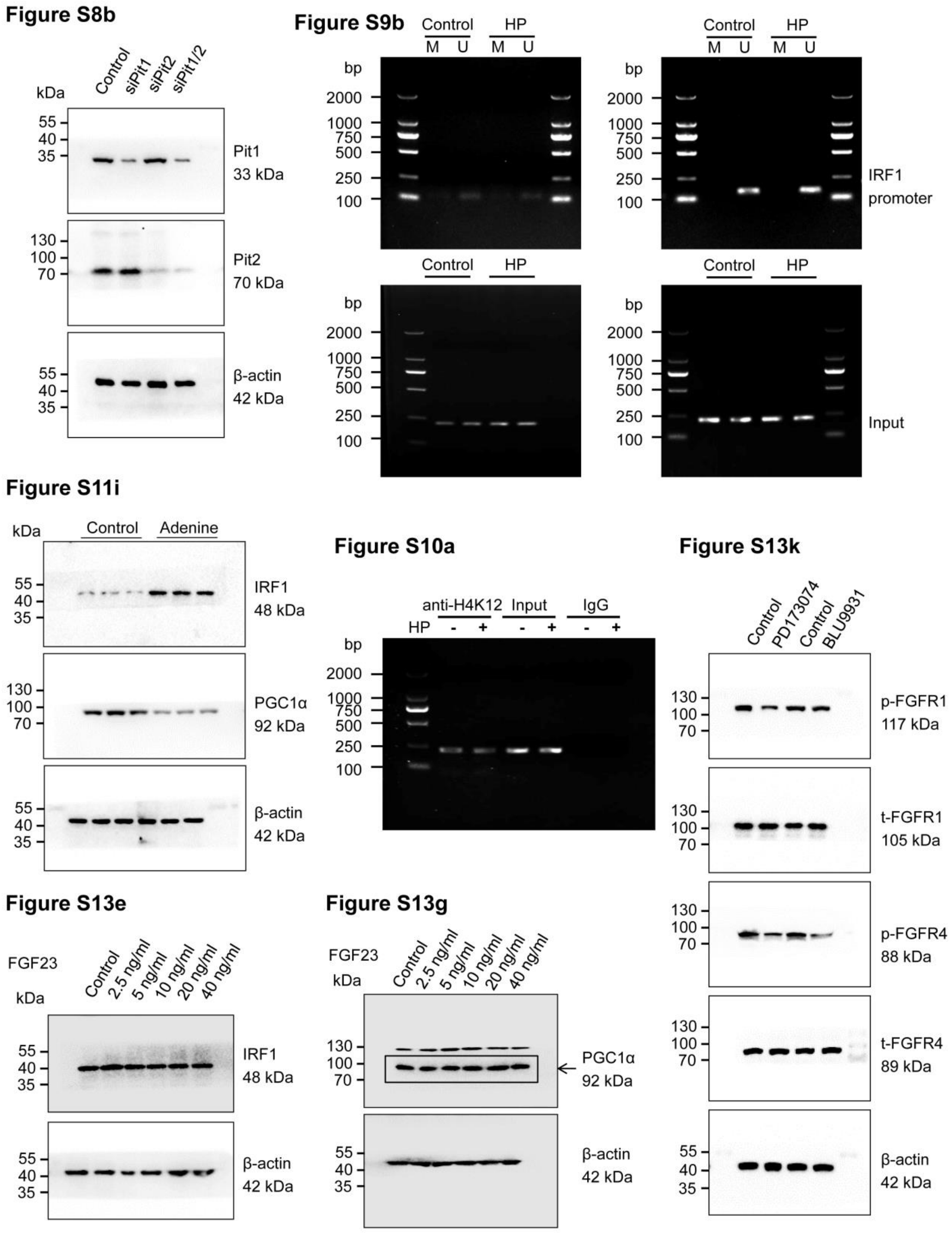

Supplementary Figure 21. Uncropped gel images for Figure S13-S16.

Figure S13l

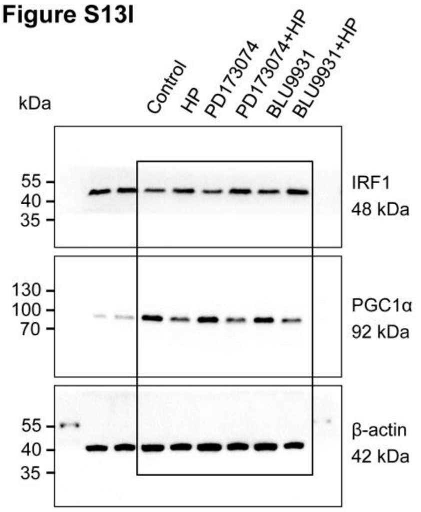

Figure S14h

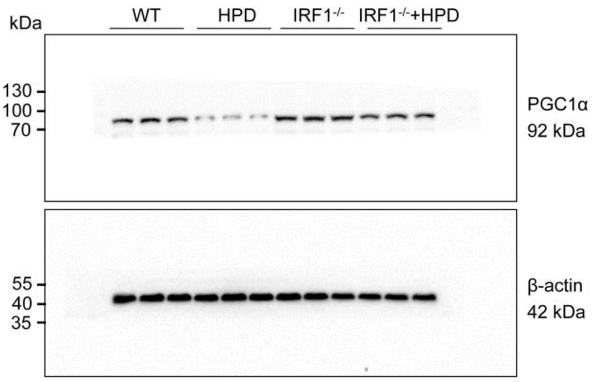

Figure S13o

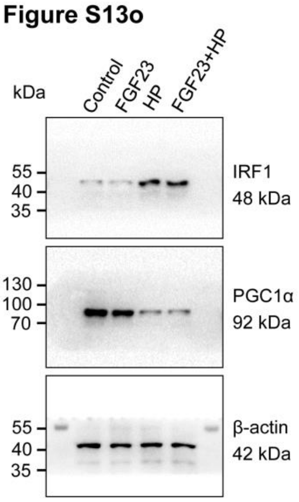

Figure S15a

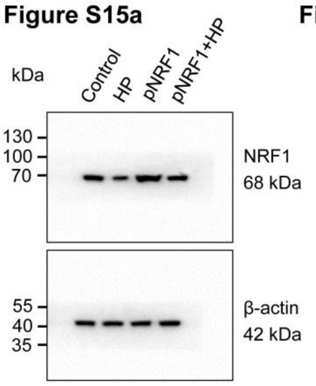

Figure S15b

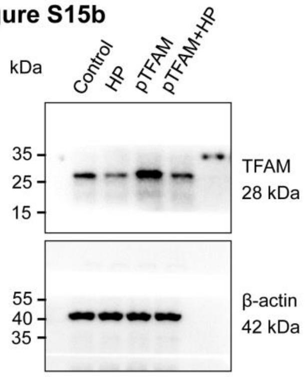

Figure S16

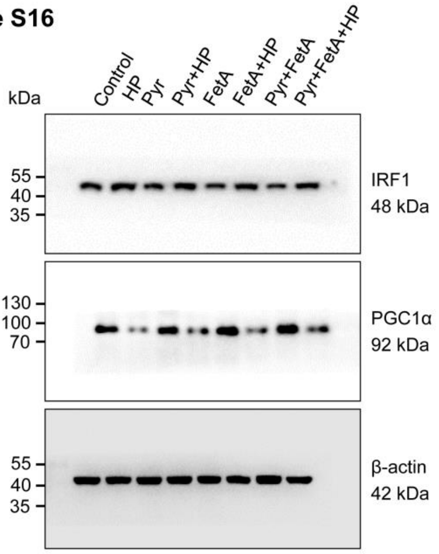

Figure S15f

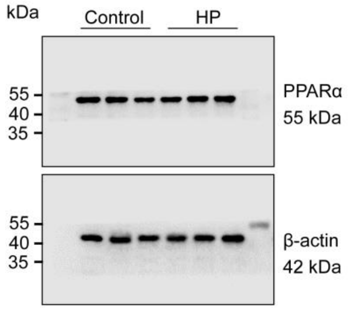

Supplementary Figure 22. Representative examples for the gating strategy.

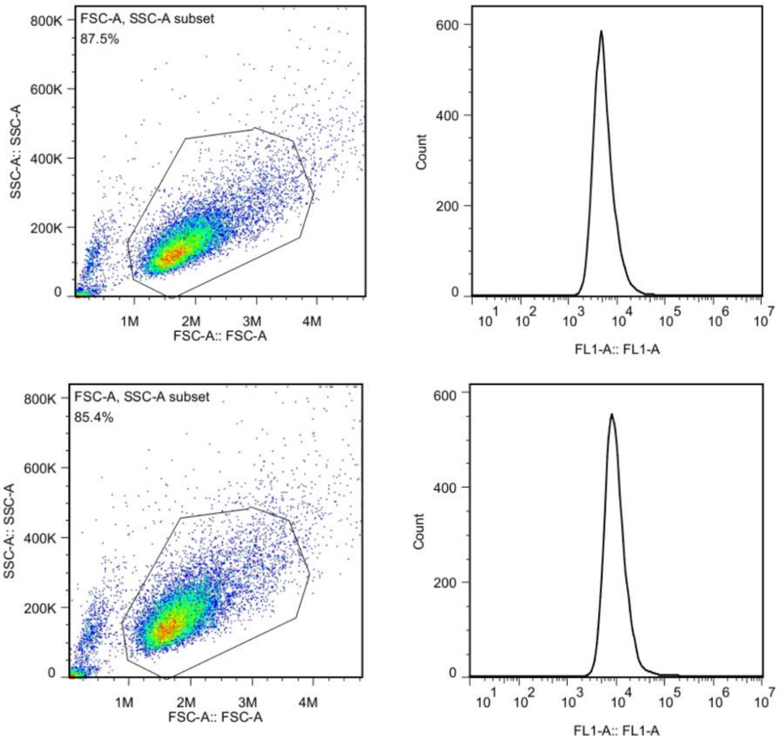

Supplementary Table 1. Putative binding sequences of IRF1 in PGC1α promoter region

Name	Score	Relative Score	Start	End	Strand	Predicted binding sites
IRF1	10.929	0.8084	-1562	-1542	+1	ATATCTTTTTTTTTTCACTTT
IRF1	7.868	0.7733	-1234	-1214	+1	AGTTGGTTTTGCATTTGGTAT
IRF1	7.866	0.7733	-974	-954	-1	CCCCCCTTTCTTTTCTTATAT
IRF1	11.231	0.8118	-632	-612	-1	TCCTTCTTTCTTTTCCCTATT
IRF1	8.442	0.7799	-253	-233	+1	GTTTCCTTTCTTTCTTCTATT

Supplementary Table 2. IRF1 binding sites in PGC1α promoter region P3 are highly conserved.

Species	Consensus sequence
Rattus norvegicus	-632 AATAGGGAAAAGAAAGAAGGA -612
Homo sapiens	-553 AATAGGGAAAAGAAAGAAGGA -533
Mus musculus	-534 AATAGGGAAAAGAAAGAAGGA -514
Bos taurus	-583 AATAGGGAAAAGAAAGAAGGA -563
Pongo abelii	-583 AATAGGGAAAAGAAAGAAGGA -563
Sus scrofa	-641 AATAGGGAAAAGAAAGAAGGA -621
Capra hircus	-645 AATAGGGAAAAGAAAGAAGGA -625
Gallus gallus	-673 AATAGGGAAAAGAAAGAAGGA -653

*The consensus IRF1 binding sites in PGC1α promoter region P3 were underlined.

Supplementary Table 3. The primer sets for mouse qPCR

Gene (mouse)	Primers	Product size
β-actin	Forward: 5′-TGTTACCAACTGGGACGACA-3′<br>Reverse: 5′-GGGGTGTTGAAGGTCTCAAA-3′	165 bp
ANF	Forward: 5′-GGAGGAGAAGATGCCGGTAGA-3′<br>Reverse: 5′-GCTTCCTCAGTCTGCTCACTCA-3′	69 bp
BNP	Forward: 5′-AAGCTGCTGGAGCTGATAAGA-3′<br>Reverse: 5′-GTTACAGCCCAAACGACTGAC-3′	222 bp
β-MHC	Forward: 5′-GTGCCAAGGGCCTGAATGAG-3′<br>Reverse: 5′-GCAAAGGCTCCAGGTCTGA-3′	84 bp
MCAD	Forward: 5′-GCAAAGGCTCCAGGTCTGA-3′<br>Reverse: 5′-ATGGCCGCCACATCAGA-3′	107 bp
CROT	Forward: 5′-GGCAAAAAGCTCACCAAGGA-3′<br>Reverse: 5′-CTGAAGAGCGAGCTGAATAAAGG-3′	63 bp
HADHB	Forward: 5′-GGGTCCGCATTCCATTTCT-3′<br>Reverse: 5′-GCCAAGTCATGTGGCATTAGG-3′	64 bp
Cpt1b	Forward: 5′-TCTAGGCAATGCCGTTCAC-3′<br>Reverse: 5′-GAGCACATGGGCACCATAC-3′	99 bp
Atp5a1	Forward: 5′-CCTTGACCTTCCTTTGCGCT-3′<br>Reverse: 5′-GCACCAACAAAGGATGACCC-3′	183 bp
Ndufa	Forward: 5′-ATCCCTTACCCTTTGCCACT-3′<br>Reverse: 5′-CCGTAGCACCTCAATGGACT-3′	198 bp

Ndufv	Forward: 5′-TGTGAGACCGTGCTAATGGA-3′<br>Reverse: 5′-CATCTCCCTTCACAAATCGG-3′	217 bp
Cytc	Forward: 5′-ACCAAATCTCCACGGTCTGTT-3′<br>Reverse: 5′-GGATTCTCCAAATACTCCATCAG-3′	126 bp
PGC1α	Forward: 5′-TCGGGAGCTGGATGGCTTGGGA-3′<br>Reverse: 5′-ACCAACCAGAGCAGCACACTCTA-3′	77 bp
TFAM	Forward: 5′-AGGCTTGGAAAAATCTGTCTC-3′<br>Reverse: 5′-TGCTCTTCCCAAGACTTCATT-3′	103 bp
ERRα	Forward: 5′-CAAGAGCATCCCAGGCTT-3′<br>Reverse: 5′-GCACTTCCATCCACACACTC-3′	77 bp
NRF1	Forward: 5′-GAACTGCCAACCACAGTCAC-3′<br>Reverse: 5′-TTTGTTCCACCTCTCCATCA-3′	70 bp
IRF1	Forward: 5′-CTTCGTCGAGGTAGGACGTG-3′<br>Reverse: 5′-CTTTGCTGCAGGAGCGATTC-3′	213 bp

Supplementary Table 4. The primer sets for rat qPCR

Gene (rat)	Primers	Product size
β-actin	Forward: 5′-CATGAAGATCAAGATCATTGCTCCT-3′<br>Reverse: 5′-GCTGATCCACATCTGCTGGAA-3′	103 bp
ANF	Forward: 5′-ATGGGCTCCTTCTCCATCAC-3′<br>Reverse: 5′-TTCATCGGTATGCTCGCTCA-3′	204 bp
BNP	Forward: 5′-TGGGAAGTCCTAGCCAGTCT-3′<br>Reverse: 5′-GATCCGGTCTATCTTCTGCC-3′	236 bp
β-MHC	Forward: 5′-TGACAGATCGGGAGAACCAG-3′<br>Reverse: 5′-CCGAACTGTCTTGGCATTGC-3′	215 bp
MCAD	Forward: 5′-TTTGCCAGAGAGGAAATAATC-3′<br>Reverse: 5′-CCAAGACCACCACAACTC-3′	134 bp
CROT	Forward: 5′-CTACCTTAAAGCCTTCTGTTGC-3′<br>Reverse: 5′-TCACAGAGGACTTGCAACCT-3′	171 bp
HADHB	Forward: 5′-GGACTTGACCTTCTTGGGGG-3′<br>Reverse: 5′-TGGGGCAGACTGTACTTGTG-3′	162 bp
Cpt1b	Forward: 5′-TCGAGTTCAGAAACGAACGC-3′<br>Reverse: 5′-GTGTGTCTCCTGGTCTCAGC-3′	114 bp
Atp5a1	Forward: 5′-TGTCATCTATGCAGGCGTCC-3′<br>Reverse: 5′-TCAGCTTTGCATCCGACTGT-3′	164 bp
Ndufa	Forward: 5′-ATGTCACGTCCTGCCATTTCT-3′<br>Reverse: 5′-ACTTGTGACCCCATTCGTCC-3′	197 bp

Ndufv	Forward: 5′-ACCTCATTTGGCTCGCTGAA-3′<br>Reverse: 5′-TCTTGTACCAATCACCCCGC-3′	106 bp
Cytc	Forward: 5′-CTTGGGCTAGAGAGCGGGA-3′<br>Reverse: 5′-TGTCTTCCGCCCAAACAGAC-3′	198 bp
PKM	Forward: 5′-GATCTGAAGTACGCCCGAGG-3′<br>Reverse: 5′-GCAGGGCCAATGGTACAGAT-3′	193 bp
HK2	Forward: 5′-AGCTTCTTTGTGTGGCTCCT-3′<br>Reverse: 5′-GGTCAACCTTCTGCACTTGG-3′	212 bp
PFKP	Forward: 5′-CAGCACCTCCTTTCCGAAGT-3′<br>Reverse: 5′-CATGCCTTGGTAACCCTCGT-3′	175 bp
PDK4	Forward: 5′-GCAGTCCTCACCAACCCTAC-3′<br>Reverse: 5′-TCTGAACCGAAGTCCAGCAG-3′	193 bp
PGC1α	Forward: 5′-GGAGCAATAAAGCAAAGAGCA-3′<br>Reverse: 5′-GTGTGAGGAGGGTCATCGTT-3′	103 bp
TFAM	Forward: 5′-CAGAGTTGTCATTGGGATTGG-3′<br>Reverse: 5′-TTCAGTGGGCAGAAGTCCAT-3′	137 bp
ERRα	Forward: 5′-GAGGTGGACCCATTGCCTTT-3′<br>Reverse: 5′-ATGGCGTACAGCTTCTCAGG-3′	137 bp
NRF1	Forward: 5′-TTGATGGACACTTGGGTAGC-3′<br>Reverse: 5′-GCCAGAAGGACTGAAAGCAG-3′	108 bp
IRF1	Forward: 5′-GCCATTCACACAGGCCGATA-3′<br>Reverse: 5′-CGGCTGGACTTGGACTTTCT-3′	212 bp

ZNF354C	Forward: 5′-CGGAGATAGAAGCGTCGTCC-3′<br>Reverse: 5′-GCTCCTGTGCTGTCTCTAGC-3′	150 bp
MEIS1	Forward: 5′-ATCAGTCCAACCGAGCAGTC-3′<br>Reverse: 5′-CTTCCCCCTGGCTTTCGATT-3′	204 bp
Tcf12	Forward: 5′-CGCTCCCGACCAAAGTGAA-3′<br>Reverse: 5′-AGCTCCTTGTCGGTCCCTAT-3′	130 bp
FOXL1	Forward: 5′-CCACAAGTTCCCGCTAGGAG-3′<br>Reverse: 5′-CAGACACTTCGGAGGACCAC-3′	207 bp
PABPC1	Forward: 5′-AATATGCCCGGTGCTATCCG-3′<br>Reverse: 5′-GACGTGGACCCATTGTCTGT-3′	136 bp
BRCA1	Forward: 5′-ACCACACAACTTAACAGGGC-3′<br>Reverse: 5′-CAGGGTCTCTGCTGGAGACTA-3′	101 bp
IGF1R	Forward: 5′-TGACACGTGGCGATCTCAAA-3′<br>Reverse: 5′-CCAGGTCTCTGTGGACGAAC-3′	165 bp
IRF2	Forward: 5′-GCCCGACATTGAGGAAGTGA-3′<br>Reverse: 5′-TCAGGACCGCATACTCAGGA-3′	221 bp
IRF3	Forward: 5′-TGGCTGCGAGTCTCAACTAC-3′<br>Reverse: 5′-GGTTTCGGGGTTCCCATGAT-3′	134 bp
IRF4	Forward: 5′-TACAGCTGCAGACTCAGTGG-3′<br>Reverse: 5′-GCAAGGGAGACTCTTCAGGAT-3′	145 bp
IRF5	Forward: 5′-GCGCTGTGCCCTTAACAAAA-3′<br>Reverse: 5′-ACTCAGGCCTGGTAGCATTC-3′	202 bp

IRF6	Forward: 5′-GTGAGCAACGGAGACAGTGA-3′<br>Reverse: 5′-ATCCCAACTTTCGGTGGCTT-3′	250 bp
IRF7	Forward: 5′-TTCAGCCGTAGGGATCTGGA-3′<br>Reverse: 5′-AAGATAAAGCGCCCTGTGCT-3′	179 bp
P300	Forward: 5′-AGGTCTGGTAGTTCCCCCAA-3′<br>Reverse: 5′-GTGCCATTGGGCTTTTGACC-3′	127 bp
CBP	Forward: 5′-TGAGAACTTGCTGGACGGAC-3′<br>Reverse: 5′-TTTGGACGCAGCATCTGGAA-3′	190 bp
PCAF	Forward: 5′-ACTGTGGGTCCTGGGTATCG-3′<br>Reverse: 5′-ATACAGAGCCCAAGCAAGCA-3′	144 bp
HAT1	Forward: 5′-CTCGGAAATGGCGGGGTTT-3′<br>Reverse: 5′-ACGAACCAGTTTCAGCTCGAT-3′	124 bp
PPARα	Forward: 5′-GTCCTCTGGTTGTCCCCTTGAG-3′<br>Reverse: 5′-GAGCACCAATCTGTGATGACAAC-3′	230 bp
FGF23	Forward: 5′-GCAACATTTTTGGATCGTATCA-3′<br>Reverse: 5′-GATGCTTCGGTGACAGGTAGA-3′	99 bp
SLC20A1<br>*(Pit1)*	Forward: 5′-TTCGTGCATTCATCCTCCGT-3′<br>Reverse: 5′-AAAACTGCACATCCCACCGA-3′	184 bp
SLC20A2<br>*(Pit2)*	Forward: 5′-TTTCCGGAGGGAACGAGAAG-3′<br>Reverse: 5′-CTGCTCTGGAAAGCGATGGA-3′	182 bp
SLC17A1	Forward: 5′-GGCAGGCCCTAAGAAAAGGT-3′<br>Reverse: 5′-CTCGCTGAGCCATGATGACA-3′	199 bp

SLC34A1	Forward: 5′-CAAACGCACTGCCAAGTACC-3′<br>Reverse: 5′-GGACGTTAACAAGCACCACG-3′	167 bp
SLC34A2	Forward: 5′-TAGCCCATAGGTGTGAGCCT-3′<br>Reverse: 5′-ACAATGTGCCAAACACGTCG-3′	167 bp
SLC34A3	Forward: 5′-CCCTCACCACACATGCAGAT-3′<br>Reverse: 5′-GTCTGTGCCTCCCTCCTCTA-3′	186 bp

Supplementary Table 5. The primer sets for rat mitochondrial DNA detection

Gene (rat)	Primers	Product size
D-Loop(mtDNA)	Forward: 5′-ATCCTCCGTGAAATCAACAA-3′<br>Reverse: 5′-CAGGACTTTGTGCTGACCTT-3′	288 bp
B2M(chDNA)	Forward: 5′-CCCAACTTCCTCAACTGCTA-3′<br>Reverse: 5′-GCTCCTTCAGAGATGACGTGT-3′	211 bp

Supplementary Table 6. The primer sets for overexpression plasmids construction

Gene (rat)	Primers (The restriction enzyme cutting sites are underlined)	Product size
PGC1α	Forward: 5′-AATTCTGCAGTCGACGGTACC ATGGCTTGGGACATGTGCAG-3o<br>Reverse: 5′-TTATCTAGATCCGGTGGATCC TTACCTGCGCAAGCTTCTCT-3o	2391 bp
IRF1	Forward: 5′-TCACTATAGGGAGACCCAAGCTT ATGCCTATCACTCGGATGCGAAT-3A<br>Reverse: 5′-ATCCGAGCTCGGTACCAAGCTT TTATGGTGCACAAGGAATAGCCTG-3C	987 bp
NRF1	Forward: 5′-GCTCGGATCCATGGAGGAACACG GAGTGACCCAAACCGAACAC-3′<br>Reverse: 5′-TAGACTCGAGTTATTTCCTTTTC AGTTGCTGTGGCGAGTTAAA-3′	1617 bp
TFAM	Forward: 5′-GCTCGGATCCATGGCGCTGTTCC GGGGAATGTGGGGCGTGCTA-3′<br>Reverse: 5′-TAGACTCGAGTTAATTCTCAGAG ATGTCTCCCGGGGGTCGCTT-3′	747 bp

PGC1α, Restriction enzyme: KpnI and BamHI, Vector: pEGFP-C1.

IRF1, Restriction enzyme: HindIII, Vector: pcDNA3.0.

NRF1 and TFAM, Restriction enzyme: BamHI and XhoI, Vector: pCDNA3.1.

Supplementary Table 7. siRNAs against IRF1 and negative control

siRNA(rat)	Primers
Negative control	Forward: 5'-UUCUCCGAACGUGUCACGUTT-3'
	Reverse: 5'-ACGUGACACGUUCGGAGAATT-3'
siIRF1-1	Forward: 5'-CCCUGGCUAGAGAUGCAGAUUAAUU-3'
	Reverse: 5'-AAUUAAUCUGCAUCUCUAGCCAGGG -3'
siIRF1-2	Forward: 5'-GCCCUCC AUUCAGGCUAUUCCUUGU-3'
	Reverse: 5'-ACAAGGAAUAGCCUGAAUGGAGGGC-3'
siPit1	Forward: 5'-GGCUCCGAAUUCAGUCAAATT-3'
	Reverse: 5'-UUUGACUGAAUUCGGAGCCTT-3'
siPit2	Forward: 5'-GAAUGACGGUCACGUUUAUTT-3'
	Reverse: 5'-AUAAACGUGACCGUCAUUCTT-3'

Supplementary Table 8. The primer sets for PCR amplification of the rat *PGC1α* promoter region

Fragment	Primers (The restriction enzyme HindIII cutting sites are underlined)
-1703 to +188	Forward: 5'-TCTGCGATCTAAGTAAGCTTGTCATCTCATTCTGTTCACTGA-3'
	Reverse: 5'-AGTACCGGAATGCCAAGCTTTACTTTCTATGTCCCCTCCTT-3'
-1524 to +188	Forward: 5'-TCTGCGATCTAAGTAAGCTTGAGAAAACTAATGTTTTCCTTC-3'
	Reverse: 5'-AGTACCGGAATGCCAAGCTTTACTTTCTATGTCCCCTCCTT-3'
-985 to +188	Forward: 5'-TCTGCGATCTAAGTAAGCTTGAAAATGCTACATATAAGAAAA-3'
	Reverse: 5'-AGTACCGGAATGCCAAGCTTTACTTTCTATGTCCCCTCCTT-3'
-719 to +188	Forward: 5'-TCTGCGATCTAAGTAAGCTTTTAGACCTAAACAAATGAGGCT-3'
	Reverse: 5'-AGTACCGGAATGCCAAGCTTTACTTTCTATGTCCCCTCCTT-3'
-348 to +188	Forward: 5'-TCTGCGATCTAAGTAAGCTTAGTTATTATATGACCAGGGCTC-3'
	Reverse: 5'-AGTACCGGAATGCCAAGCTTTACTTTCTATGTCCCCTCCTT-3'
-183 to +188	Forward:5'-TCTGCGATCTAAGTAAGCTTAGTGACGTCAGGAGTTTGAGCA-3'
	Reverse: 5'-AGTACCGGAATGCCAAGCTTTACTTTCTATGTCCCCTCCTT-3'

Supplementary Table 9. The primer sets for PCR and qPCR amplification in ChIP assays

Fragment	Primers	Product size
PGC1α(-709 to -511)	Forward: 5′-ACAAATGAGGCTGTTTGGTTGA-3′<br>Reverse: 5′-AAGCTCCTCATGTGATACGTT-3′	199 bp
PGC1α(-1711 to -1598)	Forward: 5′-AGCTGTTGGTCATCTCATTCTGT-3′<br>Reverse: 5′-TCAGACCCTGAAAAAGGGGTC-3′	114 bp
IRF1(-994 to -774)	Forward: 5′-CGATTCTGGCCACAGTTTCC-3′<br>Reverse: 5′-AACAAGGTGACCCTTCTCCC-3′	221 bp

Supplementary Table 10. The primer sets for MSP in IRF1 promoter region

Fragment	Primers	Product size
IRF1-M1	Forward: 5′-TTTTTTAAGATAGGTAAGGGGGTC-3′<br>Reverse: 5′-TTTTTTAAGATAGGTAAGGGGGTC-3′	119 bp
IRF1-U1	Forward: 5′-TTTAAGATAGGTAAGGGGGTTGG-3′<br>Reverse:55′-TTTAAGATAGGTAAGGGGGTTGG-3″	116 bp
IRF1-M2	Forward: 5′-TTTTTTCGAAATGATGAGGC-3′<br>Reverse: 5′-CGACGAAAAAATAATACGCACT-3′	150 bp
IRF1-U2	Forward: 5′-TGATTTTTTTGAAATGATGAGGT-3′<br>Reverse:55′-TGATTTTTTTGAAATGATGAGGT-3″	150 bp

*IRF1Input was the same as sequence (-994 to -774) in the IRF1 promoter region.
